# The choroid plexus acts as an immune cell reservoir and brain entry site in experimental autoimmune encephalomyelitis

**DOI:** 10.1186/s12987-023-00441-4

**Published:** 2023-06-01

**Authors:** Ivana Lazarevic, Sasha Soldati, Josephine A. Mapunda, Henriette Rudolph, Maria Rosito, Alex Cardoso de Oliveira, Gaby Enzmann, Hideaki Nishihara, Hiroshi Ishikawa, Tobias Tenenbaum, Horst Schroten, Britta Engelhardt

**Affiliations:** 1 Theodor Kocher Institute, University of Bern, Freiestrasse 1, Bern, CH-3012 Switzerland; 2 Klinik für Kinder - und Jugendmedizin, Universitätsmedizin Mannheim, Theodor-Kutzer-Ufer 1-3, 68167 Mannheim, Germany; 3 Laboratory of Clinical Regenerative Medicine, Department of Neurosurgery, University of Tsukuba, Tsukuba, Ibaraki 305-8575 Japan; 4 Present address: Clinic for Pediatrics and Adolescent Medicine, Johann Wolfgang Goethe University, Frankfurt/Main, Germany; 5 Present address: Department of Physiology and Pharmacology, Sapienza University, Rome, 00185 Italy; 6 Present address: Department of Neurotherapeutics, Yamaguchi University, Yamaguchi, 755-8505 Japan; 7 Present address: Clinic for Pediatrics and Adolescent Medicine, Sana Clinic Lichtenberg, Charité, Berlin, Germany

**Keywords:** Choroid plexus, Experimental autoimmune encephalomyelitis, CCL20, Th17 cells, T cell trafficking

## Abstract

The choroid plexus (ChP) has been suggested as an alternative central nervous system (CNS) entry site for CCR6^+^ Th17 cells during the initiation of experimental autoimmune encephalomyelitis (EAE), an animal model for multiple sclerosis (MS). To advance our understanding of the importance of the ChP in orchestrating CNS immune cell entry during neuroinflammation, we here directly compared the accumulation of CD45^+^ immune cell subsets in the ChP, the brain and spinal cord at different stages of EAE by flow cytometry. We found that the ChP harbors high numbers of CD45^int^ resident innate but also of CD45^hi^ adaptive immune cell subsets including CCR6^+^ Th17 cells. With the exception to tissue-resident myeloid cells and B cells, numbers of CD45^+^ immune cells and specifically of CD4^+^ T cells increased in the ChP prior to EAE onset and remained elevated while declining in brain and spinal cord during chronic disease. Increased numbers of ChP immune cells preceded their increase in the cerebrospinal fluid (CSF). Th17 but also other CD4^+^ effector T-cell subsets could migrate from the basolateral to the apical side of the blood-cerebrospinal fluid barrier (BCSFB) in vitro, however, diapedesis of effector Th cells including that of Th17 cells did not require interaction of CCR6 with BCSFB derived CCL20. Our data underscore the important role of the ChP as CNS immune cell entry site in the context of autoimmune neuroinflammation.

**Supplementary Information:**

The online version contains supplementary material available at 10.1186/s12987-023-00441-4.

## Introduction

The central nervous system (CNS) is shielded from the constantly changing blood milieu by the brain barriers that ensure homeostasis and intact neuronal activity. Besides the endothelial blood-brain barrier (BBB) at the level of CNS microvessels shielding the CNS parenchyma from the blood, the blood-cerebrospinal fluid barriers (BCSFB) establish barriers between the blood and the cerebrospinal fluid (CSF) filled spaces. While the BCSFB at the surfaces of brain and spinal cord between the dura mater (blood) and the CSF filled subarachnoid space is located at the arachnoid mater, the BCSFB between the blood and ventricular CSF borders the stroma of the choroid plexuses (ChP). The ChP extend as veil-like structures from the ventricular surfaces into the lumen of all four ventricles. They are responsible for the secretion of a large proportion of the CSF and control the molecular and cellular composition of the CSF [1]. The ChP are an important source of biologically active molecules involved in brain development, stem cell proliferation and differentiation, and brain repair [2]. Within the ChP stroma an extensive network of fenestrated microvessels allows for free diffusion of solutes between the blood and the ChP stroma [1]. The actual barrier that inhibits free diffusion of water-soluble molecules between the ChP stroma and the CSF is the BCSFB formed by the cuboidal epithelial cells of the ChP that enclose the ChP stroma [3] and are connected by unique parallel oriented tight junctions prohibiting paracellular diffusion [4, 5].

The brain barriers also establish the interface between the peripheral immune system and the CNS and thus strictly control immune cell trafficking into the CNS [6]. While the myeloid cells of the innate immune system establish a stereotype immune response against microbes, T and B cells of the adaptive immune system mount a highly antigen-specific immune response with immune cell memory. It is well established that the ChP stroma harbors tissue resident and thus self-renewing myeloid cells including macrophages and dendritic cells as well as blood derived innate immune cells [1, 7–9]. In contrast, CSF is rather devoid of innate immune cells with 90% of the few immune cells found within the CSF being T cells, most notably central memory T cells (T_CM_) and effector memory T cells (T_EM_) [10]. Given the absence of T cells within the healthy CNS parenchyma and their high percentage within the CSF in comparison to other sterile body fluids, a direct CSF entry of T cells across the BSCFB has been suggested. Controlling immune cell traffic between the ChP stroma and the CSF thus potentially confers on the ChP a function in neuroimmune regulation.

The molecular mechanisms controlling immune cell entry across the different brain barriers into the CNS are incompletely understood particularly for CNS immune surveillance. Much of the actual knowledge about immune cell trafficking into the CNS is derived from studies in experimental autoimmune encephalomyelitis (EAE), an animal model for multiple sclerosis (MS). In this disease context it was found that T-cell migration across the BBB is a multi-step process requiring sequential interactions of different adhesion and signaling molecules. In the absence of neuroinflammation, activated CD4^+^ Th1 cells, which produce the signature cytokine IFNγ and are epigenetically imprinted by the transcription factors T-bet [11], can cross subpial spinal cord vessels and reach perivascular sites [12] or meningeal vessels to reach the CSF drained subarachnoid space [13, 14]. While encephalitogenic Th1 cells require α4-integrins to reach the brain and spinal cord and initiate EAE, CD4^+^ Th17 cells, secreting the signalture cytokine IL-17 [15] and expressing the transcription factor retinoid‐acid-related orphan receptor γt (RORγt) [16], still reach the brain but not the spinal cord to initiate EAE in the absence of α4-integrins in an lymphocyte function-associated antigen 1 (LFA-1) dependent manner [17]. The endothelial ligands of LFA-1 and α4-integrins are intercellular adhesion molecule 1 (ICAM-1) and vascular cell adhesion molecule 1 (VCAM-1), and are upregulated at the BBB during EAE. Interestingly, ICAM1- and VCAM-1 are also expressed by ChP epithelial cells and upregulated during EAE [18, 19] and were suggested to contribute to T-cell entry into the CNS via the ChP BCSFB [20]. Th17 cells were shown to require CCR6 to initiate EAE and were found to reach the subarachnoid space of the brain via the ChP, probably by crossing the CCL20 expressing BCSFB in a CCR6-dependent manner [21].

To further explore the neuroimmune function of the ChP and specifically its role as CNS immune cell entry site the present study investigated the presence of immune cell subsets in the ChP as compared to the brain and spinal cord in healthy C67BL/6J mice and after induction of EAE by flow cytometry. We hypothesized that accumulation of immune cells in the ChP preceding their accumulation in the brain and spinal cord during EAE would be indicative for a role of the ChP as CNS immune cell entry site. Our present data show presence of high numbers of innate but also of adaptive immune cells in the ChP including CCR6^+^ Th17 cells when compared to brain and spinal cord. While immune cell numbers decreased in brains and spinal cords of mice during chronic EAE, elevated immune cell levels remained in the ChP. Increased numbers of immune cells in the ChP were followed by elevated immune cell numbers in the CSF of mice suffering from EAE. Exploring the role of CCR6 in mediating T-cell diapedesis across the BCSFB expressing CCL20 we made the surprising observation that effector T cells cross the BCSFB independent of CCR6. Taken together our data underscore a role of the ChP in CNS immunity.

## Materials and methods

### Mice

C57BL/6J mice were obtained from Janvier (Genest Saint Isle, France). 2D2 TCR MOG transgenic C57BL/6J mice (2D2) expressing a T-cell receptor recognizing MOG_aa35−55_ in the context of MHC class II (I-Ab) were obtained from Dr. Kuchroo (Boston, USA) [22]. CCR6-deficient mice were provided by Dr. Sallusto and have been described before [23]. CCR6-deficient mice were backcrossed on the C57BL/6J background for at least 6 generations. Mice were housed in individually ventilated cages under specific pathogen-free conditions. Animal procedures were performed in accordance with the Swiss legislation on the protection of animals and were approved by the veterinary office of the Kanton of Bern.

### Antibodies and reagents

All antibodies used in this study are listed in Supplementary Table 1. The CCR6 inhibitor CCX2553 was provided by ChemoCentryx, San Carlos, CA, USA.

### Induction of EAE in C57BL/6J mice

Active EAE (aEAE) was induced by subcutaneous injection of 200 µg of myelin oligodendrocyte glycoprotein peptide (MOG_aa35−55_) in 100 µl of complete Freund’s adjuvant (incomplete Freund’s adjuvant (Santa Cruz Biotechnology, USA) supplemented with 4 mg/mL non-viable, desiccated Mycobacterium tuberculosis (H37 RA, DIFCO Laboratories, Detroit, MI) into 8–12 weeks old female mice as described before [24]. 300 ng pertussis toxin from Bordetella pertussis (LuBioScience GmbH, Switzerland) per mouse was administered intraperitoneally at days 1 and 3 post immunization (p.i.). Assessment of clinical disease activity was performed as described before [24] with the following disease scores: 0 = asymptomatic, 0.5 = limp tail, 1 = hind leg weakness, 2 = hind leg paraplegia, 3 = loss of lower body control and incontinence. A total of 6 different aEAE experiments were performed dedicated for immune cell isolation from the CNS as outlined in Supplementary Fig. 1.

### Isolation of CD45^+^ cells from the choroid plexus, brain, and spinal cord

Infiltrating and resident immune cells from the brain and spinal cord of wild-type, healthy, and aEAE mice were isolated as described previously [25]. Briefly, Isoflurane-anesthetized mice were perfused with 10 mL ice-cold Dulbecco’s Phosphate Buffered Saline (DPBS). Brains and spinal cords were carefully dissected from the scull and the vertebral column, respectively and further processed without removing the leptomeninges. Tissues from 3 to 5 mice were homogenized between glass slides, pooled, and digested with collagenase VIII (0.2 mg/mL; Sigma-Aldrich, Switzerland) at 37 °C for 30 min (spinal cords) or 45 min (brains) in the presence of DNase I (1 U/mL, Roche Diagnostics, Switzerland). The digested tissue was filtered through a 100 μm pore size nylon mesh and centrifuged (10 min, RT, 200 g). The pellet was re-suspended in 5 mL 50% Percoll, overlaid with 8 mL 30% Percoll (GE Healthcare Life Sciences, Switzerland) and centrifuged at 1000 g for 30 min at 4 °C. Cells were collected from the interphase and washed twice prior to immunostainings and analysis by flow cytometry. The ChP was dissected from both lateral and fourth ventricles of the brain, resuspended in DPBS containing 400 µg/mL collagenase type IV (Worthington Biochemical Corporation), and incubated in a shaking water bath for 45 min at 37 °C before homogenization by pipetting.

### Flow cytometry

Staining of surface molecules for flow cytometry was carried out at 4 °C. Isolated cells were distributed in a 96-well plate and washed twice with FACS buffer (DPBS supplemented with 2.5% FBS and 0.1% NaN_3_) and then incubated for 15 min with 0.5 µg/ mL Fcγ III/II receptor blocking antibody (clone 2.4G2). The cells were stained with fluorescently conjugated primary antibodies for 30 min and finally fixed in 1% formaldehyde/DPBS (pH 7.4). For intracellular flow cytometry staining cells were incubated with 50 ng/mL phorbol 12-myristate 13-acetate (PMA), 1 µg/mL ionomycin and 0.7 µL/mL BD GolgiStop™ (BD Biosciences, Switzerland) for 5 h at 37 °C. All further steps were performed at room temperature (RT). Stimulated cells were fixed in BD Cytofix™ Buffer (BD Biosciences, Switzerland) for 15 min, followed by permeabilization with BD Perm/Wash™ Buffer (BD Biosciences, Switzerland) for another 15 min and stained with the respective antibodies for 30 min. All washings steps throughout the staining procedure were done with FACS buffer. Samples were measured with either a FACSCalibur or BD Biosciences LSRII flow cytometer and analyzed using FlowJo v10.7 and v10.8 (BD Life Sciences). Quantification of CD45^+^ cells was performed with reference beads (BD Trucount tubes), according to the manufacturer’s instructions.

### Detection of CSF immune cells by cytospin

Mice deeply anesthetized with isoflurane were perfused with 10 mL DPBS via the left ventricle of the heart and placed under the dissection microscope. The subcutaneous tissue and muscles of the neck were pushed aside to expose the cisterna magna. 5 µL CSF/mouse was drawn by puncture of the cisterna magna with a glass capillary tube as described in [26]. 15 µL of CSF (pooled from 3 mice) were diluted with FBS/DPBS (2:1) solution to an end volume of 100 µL. The diluted CSF was filled into a cytofunnel (Thermo Fisher Scientific Inc.), fixed on FBS/DPBS (2:1) precoated glass slide and centrifuged at 200x g for 5 min. After 5 min air-drying the slides were fixed with 1% PFA for 10 min and CSF immune cells concentrated on the slide were stained for CD45 and CD4.

### Immunohistology

Mice were anaesthetized with isoflurane (Baxter, Arovet AG, Switzerland) and perfused with cold DPBS. Brains and spinal cords were dissected, embedded in Tissue-Tek (OCT compound, Sysmex Digitana AG, Switzerland) and snap frozen in a dry ice/isopentane bath (2-Methylbutane, Grogg Chemie AG, Switzerland). Cryostat Sect. (6 μm) were air dried overnight, fixed in acetone at -20 °C for 10 min, and stained for immunohistology using a three strep immunoperoxidase technique as described before [27].

### Isolation and culture of primary mouse choroid plexus epithelial cells

Monolayers of primary mouse ChP epithelial cells (pmCPECs) were obtained and cultured exactly as described by us before [28].

### Immunofluorescence stainings of pmCPECs

Confluent cell layers grown on Transwell filters (Corning) were gently washed with warm DPBS and subsequently fixed with 1% paraformaldehyde (PFA, MERCK) in DPBS at RT for 10 min. After fixation, the cells were washed 5 times with DPBS and incubated in blocking solution (5% skimmed milk (Rapilait, Migros), 0.3% Triton-x-100 (Schweizerhall Batch Nr. A11021), 0.04% NaN_3_ (Fluka) in TBS pH 7.4) for 20 min at RT. Subsequently, first antibody in blocking solution was added for 1 h at RT. The primary antibody was removed from the cell layer by washing 5 times with DPBS. Secondary antibody was incubated for 45 min at RT. After washing with DPBS the cells were incubated with 4′, 6-Diamidin-2-phenylindol (DAPI, 1 µg/mL, Axonlab) in DPBS for 2 min at RT. After 3 times washing with DPBS, cells carrying filters were cut out from the inserts, plated on glass-slides, and mounted with Mowiol (Aldrich). ICAM-1 and VCAM-1 staining was performed on living cells. Therefore, unfixed cells were directly incubated with the hybridoma culture supernatants for 20 min without a prior blocking step. After washing, the cells were fixed and incubated with the secondary antibody for 20 min at RT in the dark.

### On cell western

On cell western for a quantitative analysis of cell surface protein expression was performed as previously described [29]. In brief, pmCPECs grown to confluence in a 96-well clear flat-bottom plate (Greiner Bio-One, Monroe, NC) were stimulated with IFNγ (100 U/mL, R&D Systems) and TNFα (10 ng/mL, R&D Systems) for 24 and 48 h. After washing, cells were incubated for 15 min with hybridoma culture supernatant containing antibodies against ICAM-1 or VCAM-1 or isotype control (Supplementary Table 1). The pmCPECs were washed 3 times with DMEM supplemented with 5% FCS and 25 mM Hepes and incubated with the second stage antibody for 15 min. After washing, cells were imaged with the Odyssey Infrared Imaging System (LI-COR Biosciences, Bad Homburg, Germany). Values were normalized to the non-stimulated condition after subtracting the background value of the isotype control.

### Permeability of pmCPEC monolayers

Monolayers of pmCPECs were stimulated with IFNγ (100 U/mL, R&D Systems) or TNFα (10 ng/mL, R&D Systems) and permeability for Lucifer Yellow (Sigma) was assessed as described previously [30].

### In vitro polarization of mouse T-cell subsets

Lymph nodes and spleen from MOG_35-55_-specific T cell receptor transgenic (2D2) mice and CCR6-deficient 2D2 mice (2D2 CCR6 KO) mice were homogenized with a Wheaton loose homogenizer. The suspension was centrifuged for 8 min at 250 x g, erythrocytes were lysed with 0.83% ammonium chloride/Tris hydrochloride (9:1) and the washed cell suspension was filtered through a 100 µM nylon mesh, centrifuged for 8 min at 250 x g, and the pellet resuspended in 10 mL of restimulation medium (RM, 10% fetal calf serum (FCS, Gibco), 1% non-essential amino acids (Gibco), 1% Na-Pyruvate (Gibco), 1% Penicillin and Streptomycin (Gibco), 2% Glutamine (Gibco), 0.05 mM β-Mercaptoethanol (Merck), in RPMI 1640 (Gibco)) for counting. The EasySep negative selection kit (Stem Cell) was used for CD4^+^ T cell isolation according to the manufacturer’s instructions. 2 × 10^4^/well CD4^+^ T cells were plated onto NUNC 96-well plates (Thermo Scientific) coated with 2 µg/mL anti CD3 and 2 µg/mL anti CD28 antibodies. The cells were polarized as follows with recombinant mouse cytokines: Th0 cells: no cytokines, Th1 cells: 5 ng/mL IL-12 (Peprotech), Th2 cells: 4 ng/mL IL-4 (Amimed Produkte AG), Th17 cells: 20 ng/mL IL-6 (R&D Systems), 1 ng/mL TGFβ (R&D Systems), 10 ng/mL IL-1β (Peprotech). The plate was incubated for 5 days in RM supplemented with 1% IL-2 containing supernatant from IL2 × 63AgO cells at 37 °C and 7% CO_2_.

### T-cell migration across pmCPEC monolayers in vitro

The migration rate of CD4^+^ in vitro polarized T cells and freshly isolated naïve T cells across the ‘inverted’ pmCPEC monolayers was measured as previously described [28]. The medium in the lower compartment of the filter system contained either CXCL12 (100 ng/mL, R&D Systems) or the pmCPECs on the filters were stimulated from the bottom with TNFα (10 ng/mL, R&D Systems) or IFNy (10 ng/mL, R&D Systems) for 24 h prior to the transmigration assay. TNFα or IFNy was removed from the pmCPECs by washing the cell layers with migration assay medium (MAM: 90% DMEM, 5% FCS, 2% 25mM HEPES, 2% glutamine) followed by addition of 4 × 10^5^ T lymphocytes into the filter inserts. Assays were performed in triplicates. After 8 h, the transmigrated T cells were collected from the lower compartment and were stained with PE-conjugated rat anti-mouse CD4 (Biolegend). Both, the number of transmigrated CD4^+^ T cells and the number of cells in input samples were counted by using Attune NxT Flow Cytometer (Thermofisher Scientific, Switzerland) by gating on PE-positive cells. The percentage of transmigrated T cells was assessed referring to the inputs as 100% as described [28].

### Isolation of human CD4^+^ effector/memory T-cell subsets

Human CD4^+^ T cells were isolated from buffy coats of healthy blood donors obtained from the Swiss Red Cross. Human primary cell protocols were approved by the Swiss Federal Office of Public Health. Informed consent from blood donors was approved by the local ethical committee (Comitato Etico Cantonale, http://www.ti.ch/CE, authorization n. CE3428). T cells were isolated by fluorescence activated cell sorting and expanded in vitro as previously described [31–33] and subsequently recharacterized by flow cytometry analysis.

### T-cell migration across the human in vitro BCSFB

Transmigration of different T lymphocyte populations was assessed according to a previously described but slightly modified method [34]. HIBCPP were seeded on inverted Millicell® filters with 5 μm pore size (Millipore) and grown until confluency. Establishment of a proper barrier function was evaluated by TEER values above 250 Ohm/cm^2^. Cell culture inserts were then placed in 24-well plates containing 600 µl Migration Assay Medium (MAM; DMEM with 5% FBS, 25 mM HEPES, 2% glutamine) in the presence or absence of CCL20 (MIP-3a) (500 ng/mL, Stem Cell). Human T cells were prelabelled with 1µM CellTrackerTM Green (CMFDA Dye, Life technologies) for 30 min at 37 °C before starting of the experiment. In addition, T cells were incubated with 200 nM CCR6 antagonist (CCX2553, ChemoCentryx, USA) or vehicle control (DMSO) for 20 min prior to the transmigration assay. Triplicates of 1.5 × 10^5^ T lymphocytes were added into the filter inserts in a total volume of 200 µl MAM and incubated for 8 h.

Migrated T cells were collected from the bottom compartment and counted using the Attune NxT Flow Cytometer (Thermofisher Scientific, Switzerland) by gating on CMFDA^+^ cells. The percentage of transmigrated T cells was assessed referring to the inputs as 100%.

### CCL20 secretion by pmCPECs and HIBCPP

Polarized secretion of CCL20 by pmCPECs and HIBCPPs to the apical and basolateral side was determined by ELISA of culture media from non-stimulated or stimulated cell monolayers (HIBCPP: 16 h, 1ng/mL TNFα; 20 IU/mL IFNy; pmCPECs: 16 h, 10 ng/mL TNFα) grown on Millicell® filter inserts using the human MIP-3a (CCL20) ELISA kit (Thermo Scientific) and the mouse CCL20/MIP-3a Quantine ELISA kit (R&D systems) and following the manufacturer’s instructions. Absorbance was measured using Tecan Infinite M1000 Pro microplate reader (Thermo Scientific). The total amount of CCL20 (pg) secreted to each side of the pmCPECs and HIBCPPs monolayers was calculated from the concentrations obtained (pg/mL) by ELISA based on the volumes of culture medium in the upper/basolateral (0.5 mL) and lower/apical (1mL) compartments.

### T-cell chemotaxis

Chemotaxis of human T cells towards CCL20 was assessed in triplicates by allowing 1.5 × 10^5^ T cells to migrate for 2 h at 37 °C across laminin (50 µg/mL, from Engelbreth-Holm-Swarm murine sarcoma basement membrane, Sigma) coated 5.0 μm pore Millicell® filters (pore density 2.0 × 10^6^ pores per cm^2^, growth area 0.33 cm^2^) in the presence or absence of human recombinant CCL20 (MIP-3a) (500 ng/mL, Stem Cell) in the bottom compartment. Human T cells were prelabelled with 1µM CellTrackerTM Green (CMFDA Dye, Life technologies) for 30 min at 37 °C before starting of the experiment.

Human T cells were incubated with 200 nM CCR6 antagonist (CCX2553, ChemoCentryx, USA) or vehicle control (DMSO) for 20 min prior to the chemotaxis assay. Preincubation of human T cells with 50 ng/mL of pertussis toxin (PTX) (List Biological Laboratories, Campbell, CA, USA) or PTX-B oligomer (PTX-B) (Calbiochem, San Diego, CA, USA) lacking the enzymatic activity of PTX were used as a control. Migrated T cells were collected from the bottom compartment and counted using the Attune NxT Flow Cytometer (Thermofisher Scientific, Switzerland) by gating on CMFDA^+^ cells. The percentage of transmigrated T cells was assessed referring to the inputs as 100%.

### Statistical analysis

Results are shown as means ± SD or SEM. Comparison between multiple groups was assessed by one-way ANOVA followed by the Tukey’s multiple comparison test, while a two-way ANOVA followed by Šídák’s multiple comparisons test was instead used when multiple groups and conditions were compared. Statistical analyses comprising calculation of degrees of freedom were performed using GraphPad Prism 9 software (GraphPad software, La Jolla, CA, USA) (p < 0.05 = *, p < 0.01 = **, p < 0.001 = ***, p < 0.0001 = ****).

## Results

### CD45^+^ immune cells reside in the choroid plexus irrespective of neuroinflammation

**Fig. 1:**
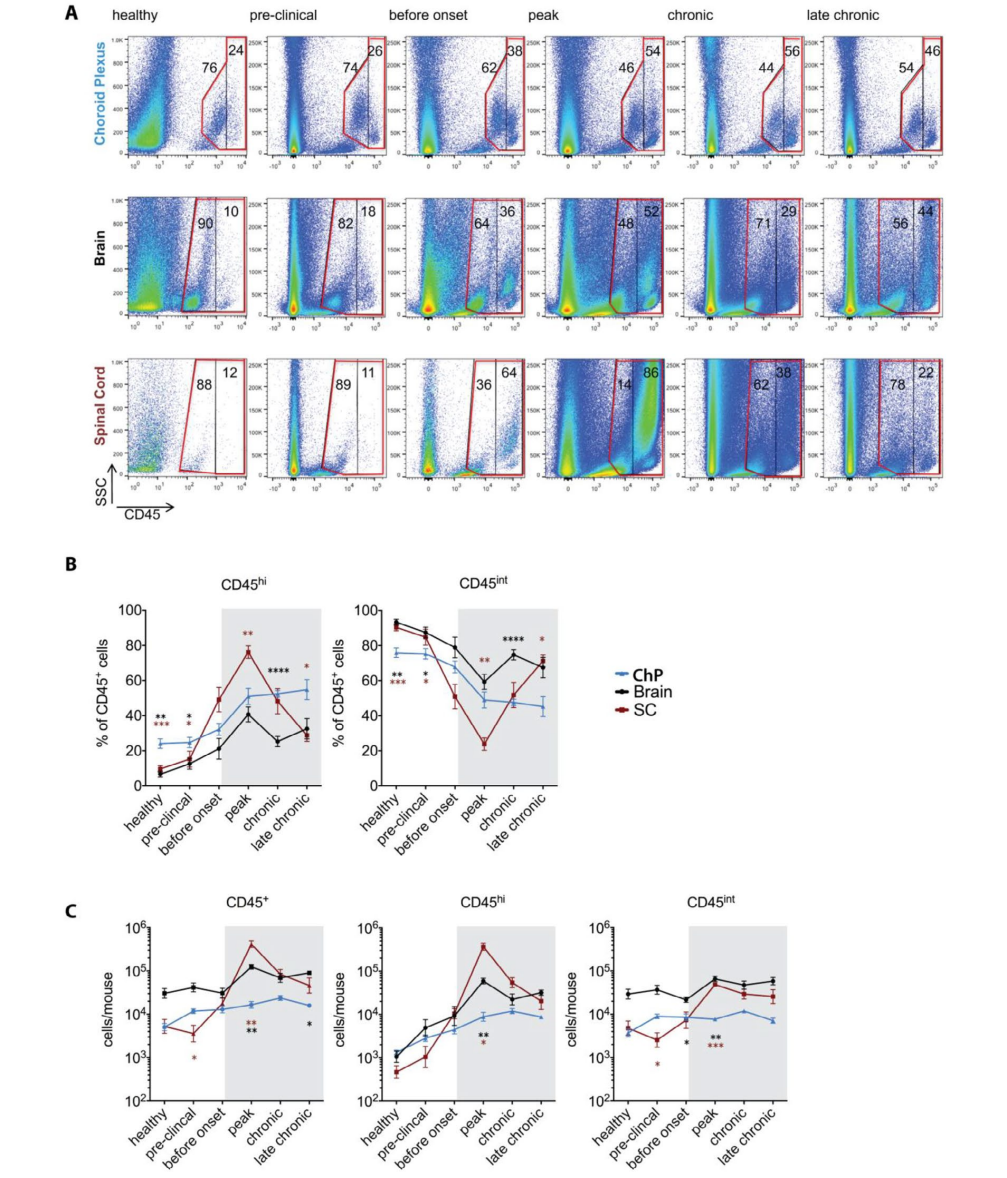
CD45^+^ immune cell subsets change after induction of aEAE in brain, spinal cord, and choroid plexus. Immune cells were isolated from the choroid plexus (ChP) of the fourth and both lateral ventricles, brains, and spinal cords (SC) of healthy C57BL/6J mice and C57BL/6J mice suffering from aEAE at the different disease time points as outlined in Supplementary Fig. 1 and analyzed by multi-color flow cytometry for CD45^+^ immune cell subsets. **(A)** Representative dot plots (SSC versus CD45) show the ungated events acquired from ChP, brain and SC and the gating strategy for CD45^+^ immune cells. Gate bordered by the red line depicts 100% of CD45^+^ immune cells, which is divided by the black line into two gates for CD45^int^ and CD45^hi^ immune cells subsets. Numbers within the gates depict the percentages of CD45^int^ and CD45^hi^ immune cells, respectively. **(B)** Quantification of the percentages of CD45^int^ and CD45^hi^ immune cell subsets in the ChP, brain and SC during EAE. **(C)** Absolute numbers of CD45^+^ as well as CD45^int^ and CD45^hi^ immune cell subsets per mouse in the ChP, brain and SC in healthy C57BL/6J mice and at the investigated time points during aEAE are shown. The graphs in B and C show means ± SEM of six independent EAE experiments as outlined in Supplementary Fig. 1B. Significant differences between ChP and SC or ChP and brain are shown with brown and black stars, respectively. Statistical analysis: one-way ANOVA (p < 0.05 = *, p < 0.01 = **, p < 0.001 = ***, p < 0.0001 = ****)

To determine the role of the choroid plexus (ChP) in immune cell entry into the CNS during autoimmune neuroinflammation, we first examined the kinetics of immune cell accumulation in the ChP, brain and spinal cord (SC) during health as well as before and during the development of MOG_aa35−55_ induced aEAE in C57BL/6J mice. To this end, we isolated immune cells from the ChP of the fourth and both lateral ventricles, the brain, and the SC of healthy mice and of mice at 5 stages after the induction of aEAE (Supplementary Fig. 1) and analyzed the CD45^+^ immune cell subsets by multi-color flow cytometry (Fig. 1).

In ChP, brain and SC of healthy mice, more than 75% of the CD45 expressing cells were CD45^int^ resident myeloid cells (Fig. 1B). The percentage of CD45^+^ cells that were CD45^hi^ was significantly higher in the ChP (24.1 ± 2.7%, mean ± SEM) of healthy mice compared to brain (6.6 ± 1.7%, p = 0.0019) and SC (9.7 ± 1.9%, p = 0.0006), supporting the notion that under steady state CD45^+^ circulating cells extravasate into the ChP but not into the brain and SC (Fig. 1B). At the peak of aEAE we found a significant increase of the percentage of CD45^hi^ infiltrating immune cells in the ChP (p = 0.0001), brain (p < 0.0001) and SC (p < 0.0001), when compared to healthy mice (Fig. 1B). Generally, the percentage of CD45^hi^ infiltrating cells in the ChP and the brain gradually increased until the peak of aEAE, whereas in the SC there was a marked increase in the percentage of CD45^hi^ inflammatory cells between the pre-clinical and peak stage (p < 0.0001) (Fig. 1B). The percentage of CD45^hi^ cells was highest during the peak of aEAE in ChP, brain and most prominent in the SC and declined after the peak in the latter two. Interestingly, the percentage of the CD45^hi^ infiltrating cells in ChP remained high and stable during the chronic phase of aEAE (Fig. 1B).

Determining next the absolute numbers of the CD45^+^ immune cells present in the brain and SC as compared to the ChP, we found the absolute numbers of CD45^hi^ immune cells in the healthy ChP combined from both lateral and the 4th ventricles (1.3 × 10^3^ ± 0.2 × 10^3^ /mouse; mean ± SEM) to be comparable to the number of CD45^hi^ cells in a healthy brain (1.1 × 10^3^ ± 0.4 × 10^3^ /mouse, p = 0.729), and more than two times higher than in the healthy SC (0.5 × 10^3^ ± 0.2 × 10^3^ /mouse, p = 0.126) (Fig. 1C). This large number of CD45^hi^ immune cells present in the healthy ChP points to an important immune function of the ChP in CNS immunosurveillance, especially when considering the small volume of the ChP compared to the brain and the SC. Numbers of CD45^hi^ immune cells further increased in the ChP, brain and SC at each stage of aEAE when compared to healthy mice, reaching a significant difference at the peak of disease (Fig. 1C). The increase was most prominent in the SC, where we found 789 times more CD45^hi^ cells in peak-stage mice than in healthy mice (peak 3.6 × 10^5^ ± 7.8 × 10^4^ /mouse; healthy 4.7 × 10^2^ ± 1.8 × 10^2^ /mouse, p < 0.0001) and less pronounced increases in brain (56 times increase, p < 0.0001) and ChP (7 times increase, p = 0.0065). While after EAE peak numbers of CD45^hi^ immune cells decreased in brain and SC during chronic and late chronic EAE, they rather plateaued in the ChP (Fig. 1A-C).

During the entire course of aEAE, there was only a slight increase in the numbers of CD45^int^ resident myeloid cells in the ChP and brain when compared to the numbers of CD45^int^ resident myeloid cells found in the ChP (3.7 × 10^3^ ± 0.9 × 10^3^ /mouse) and brain (2.9 × 10^4^ ± 0.9 × 10^4^ /mouse) of healthy animals. Within the SC, however, we found 10 times more CD45^int^ cells at the peak of clinical aEAE, than in the healthy SC (4.8 × 10^3^ ± 2.2 × 10^3^ /mouse, p < 0.0001).

In summary, we found high numbers of CD45^+^ immune cells in the ChP of healthy mice with the majority identified as CD45^int^ resident immune cells. While numbers of CD45^hi^ infiltrating immune cells in ChP massively increased during the development of aEAE and remained high during the chronic stages of the disease, numbers of CD45^hi^ infiltrating immune dropped in the brain and SC after the peak of EAE. These observations underscore an important role of the ChP in autoimmune neuroinflammation.

### The ChP harbors high numbers of CD4^+^ and CD8^+^ T cells that increase during aEAE progression

As EAE is a CD4^+^ T-cell mediated disease, an early accumulation of especially CD45^hi^ CD4^+^ T cells in the ChP prior to their accumulation within the brain and SC and prior to disease onset would support the hypothesis of the ChP being an initial site for CD4^+^ T-cell entry into the CNS. Therefore, we next analyzed the presence of CD45^hi^CD3^+^CD4^+^ and CD45^hi^CD3^+^CD8^+^ T cells in the ChP, SC and brain prior and during aEAE by multi-color flow cytometry (Supplementary Fig. 2).

**Fig. 2:**
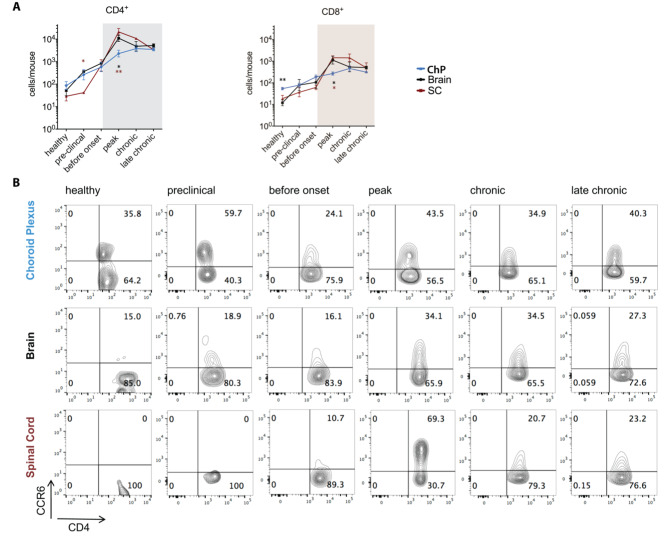
The choroid plexus harbors high numbers of CD4^+^ and CD8^+^ T cells as well as CD4^+^CCR6^+^ T cells when compared to brain and spinal cord. **(A)** Absolute numbers of CD45^hi^ CD4^+^ and CD45^hi^ CD8^+^ T cells per mouse in the choroid plexus (ChP), brain and spinal cord (SC) of healthy mice and mice during progression of aEAE as determined by multi-color flow cytometry. The graphs show means ± SEM of six independent experiments as outlined in Supplementary Fig. 1. **(B)** Contour plots for CD4 and CCR6 of CD45^hi^ immune cells isolated from ChP, brain and SC are shown. Numbers depict percentages of cells detected in the respective quadrant. Data are representative for two experiments (healthy, pre-clinical, before onset) and three experiments (peak, chronic and late chronic stages). Significant differences between ChP and SC or ChP and brain are shown in brown and black stars, respectively. Statistical analysis: one-way ANOVA (p < 0.05 = *, p < 0.01 = **, p < 0.001 = ***, p < 0.0001 = ****)

In healthy mice, CD45^hi^CD3^+^ T cells constituted 5–10% of all CD45^hi^ cells in ChP, brain and SC. In healthy mice the absolute numbers of CD4^+^ T cells and CD8^+^ T cells were higher in the ChP (CD4^+^ 86.2 ± 19 /mouse; CD8^+^ 54 ± 8 /mouse; mean ± SEM), when compared to the SC (CD4^+^ 29 ± 8 /mouse, p = 0.146; CD8^+^ 19 ± 8 /mouse; p = 0.157) and the brain (CD4^+^ 52 ± 17 /mouse, p = 0.589; CD8^+^ 11 ± 2 /mouse, p = 0.007) (Fig. 2A). Interestingly, the numbers of CD4^+^ but not of CD8^+^ T cells in the ChP were significantly higher than in the SC at the pre-clinical stage of the disease (CD4^+^ T cells: p = 0.046; CD8^+^ T cells: p = 0.55). Furthermore, in the ChP the number of CD4^+^ but not of CD8^+^ T cells were already significantly higher at the pre-clinical EAE stage when compared to the healthy controls (CD4^+^ T cells: p = 0.029; CD8^+^: p = 0.243). This supports the notion that in vivo activation of CD4^+^ T cells by immunization with MOG_35 − 55_ in CFA leads to their early recruitment into the ChP stroma.

The numbers of CD4^+^ and CD8^+^ T cells in the ChP continuously increased with reaching the highest significantly different numbers compared to the healthy condition at the peak and chronic phase of the disease for CD4^+^ T cells (p = 0.0008) and CD8^+^ T cells (p = 0.0033), respectively, and plateauing at the chronic phase of EAE. Similar to the kinetics observed for the accumulation of the CD45^hi^ infiltrating immune cells described above, numbers of infiltrating CD4^+^ and the CD8^+^ T cells in brain and SC increased shortly before and until the peak of aEAE but subsequently declined again during the chronic disease stages (Fig. 2A). CD4^+^ T helper cells outnumbered CD8^+^ cytotoxic T cells in ChP, brain and SC at all investigated timepoints during EAE, even more than 10-fold at the peak of EAE (Fig. 2A).

We have previously shown that encephalitogenic CCR6^+^ Th17 cells initiate EAE by entering the brain via the ChP in a CCR6/CCL20 dependent manner [21]. Comparing the accumulation of CCR6^+^CD4^+^ T cells in the ChP, brain and SC prior and during EAE by flow cytometry (Fig. 2B and Supplementary Fig. 3) we observed that already in healthy mice the ChP but not the brain and SC harbor a high fraction of CCR6^+^CD4^+^ T cells (Fig. 2B). The fraction of ChP CCR6^+^CD4^+^ T cells transiently increased to almost 60% at preclinical EAE and readily declined before EAE onset to increase again during ongoing EAE (Fig. 2B). The potential role of the ChP as CNS entry site for CCR6^+^CD4^+^ T cells was further underscored by the kinetics of CCR6^+^CD4^+^ T-cell accumulation observed in the brain, which followed that of the ChP, however, only reaching a fraction of 35% of CCR6^+^CD4^+^ T cells amongst the infiltrating CD4^+^ T cells. In contrast, accumulation of CCR6^+^CD4^+^ T cells in the SC was transient with a sharp rise in CCR6^+^CD4^+^ T cells during the peak followed by a decline during chronic EAE (Fig. 2B).

The cytokines IFNγ and IL-17 in combination with GM-CSF are signature cytokines for encephalitogenic Th1 and Th17 cells, respectively. Therefore, we next investigated the cytokine profile of CD3^+^ T cells by flow cytometry. In heathy mice most T cells detected in the ChP, SC and brain stained positive for IFNγ with only the ChP harboring IL-17^+^IFNg^neg^GM-CSF^neg^CD3^+^ T cells, the fraction of which increased during EAE (Supplementary Fig. 3). In contrast, the fraction of IFNy^+^IL-17^neg^CD3^+^ T cells in the ChP did not change during EAE. Interestingly, expression of GM-CSF was readily observed in IL-17^+^ and IFNy^+^ CD3^+^ T cells in the brain and SC already before EAE onset but only at the peak of EAE on very few CD3^+^ T cells in the ChP. Similarly, co-expression of IL-17 and IFNγ as described for Th1* cells was only observed in the brain and SC but not in ChP CD3^+^ T cells starting before onset of EAE (Supplementary Fig. 3) underscoring the potential plasticity of CNS infiltrating Th1 and Th17 cells with respect to expression of cytokines [35–37].

Considering its small volume when compared to the brain and SC, the ChP thus harbors high numbers of CD4^+^ and CD8^+^ T cells. Presence of a significant fraction of CCR6^+^CD4^+^ T cells in the ChP, but not in the brain and SC, supports the notion of CCR6^+^CD4^+^ T cells preferentially home to the ChP from where they might enter the CNS.

### B cells do not accumulate in the ChP during EAE

**Fig. 3:**
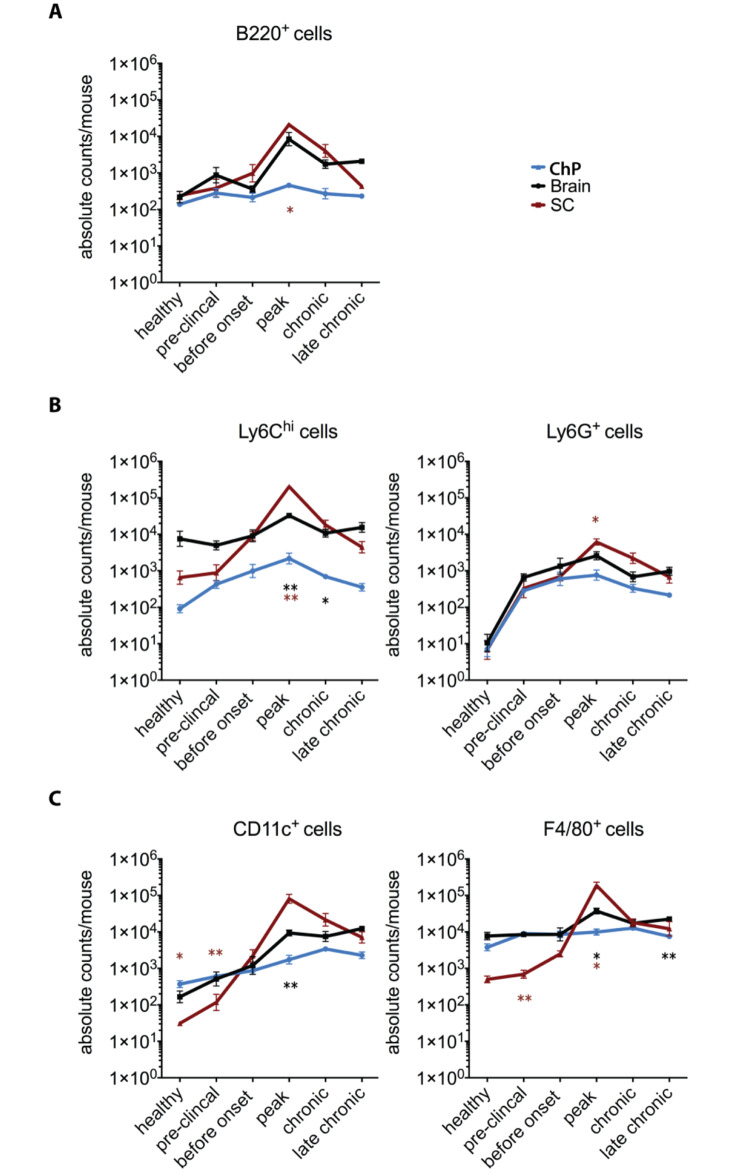
The composition of the immune cells in choroid plexus, brain, and spinal cord during aEAE progression. Absolute numbers of B220^+^ B cells **(A)** and CD11b^+^ myeloid subsets including Ly6G^+^ neutrophils, Ly6C^hi^ monocytes **(B)**, CD11c^+^ dendritic cells and F4/80^+^ macrophages **(C)** per mouse in the choroid plexus (ChP), brain and spinal cord (SC) of healthy mice and mice suffering from aEAE, were acquired by flow cytometry as shown. The graphs show means ± SEM of six independent experiments. Significant differences between ChP and SC or ChP and brain are shown in brown and black stars, respectively. Statistical analysis: **(A-C)** one-way ANOVA (p < 0.05 = *, p < 0.01 = **, p < 0.001 = ***, p < 0.0001 = ****)

In addition to T cells, B cells and myeloid cells contribute to EAE pathogenesis [38]. Thus, we next explored accumulation of B220^+^ B-cells and CD11b^+^ myeloid subsets including Ly6G^+^ neutrophils, Ly6C^hi^ monocytes, CD11c^+^ dendritic cells and F4/80^+^ macrophages in ChP, brain and SC during EAE (Fig. 3 and Supplementary Fig. 4).

Although in healthy mice the ChP was found to harbor similar numbers of B220^+^ B cells when compared to brain and SC (Fig. 3A), B220^+^ B cells only accumulated in the brain and SC but not in the ChP during EAE, as shown by a significantly higher number of B220^+^ B cells in the SC at peak of the disease when compared to the ChP (p = 0.016). Furthermore, the ChP of healthy mice was found to harbor higher numbers of CD45^+^CD11b^+^CD11c^+^ dendritic cells and CD45^+^CD11b^+^F4/80^+^ macrophages when compared to brain (CD45^+^CD11b^+^CD11c^+^: p = 0.059, CD45^+^CD11b^+^F4/80^+^: p = 0.298) and SC (CD45^+^CD11b^+^CD11c^+^: p = 0.0395, CD45^+^CD11b^+^F4/80^+^: p = 0.142) (Fig. 3C and Supplementary Fig. 4B). During EAE the numbers of CD45^+^Ly6C^hi^ monocytes as well as CD45^+^Ly6G^+^ neutrophils increased with similar kinetics in the ChP and in the brain and SC, reaching significant difference at the peak of the disease when compared to healthy condition (Fig. 3B). In contrast, the numbers of CD45^+^F4/80^+^ macrophages remained constant in the ChP during EAE but transiently increased in SC and to a lesser degree also in brain (Fig. 3C). In healthy mice, CD45^int^CD11b^+^ resident myeloid cells outnumbered CD45^hi^CD11b^+^ myeloid cells in each tissue (Supplementary Fig. 4A). While the overall numbers of CD45^int^CD11b^+^ resident myeloid cells remained significantly unchanged in the ChP and brain during EAE, we found overall increased numbers of CD45^int^CD11b^+^CD11c^+^ myeloid cells in the brain and SC but not in the ChP (Supplementary Fig. 4A). In contrast, there was a marked increase in CD45^hi^CD11b^+^CD11c^+^ cells during aEAE in the brain and the SC (57-fold: p = 0.0008, and 2617-fold: p = 0.0013, respectively; healthy compared to EAE peak), and a 3-fold (p = 0.091) and 9-fold (p < 0.0001) increase in the ChP at peak and chronic phase of EAE respectively compared to healthy condition (Fig. 3C and Supplementary Fig. 4A, B).

Taken together, we found that the ChP harbors high numbers of B220^+^ B cells in addition to CD45^int^CD11b^+^CD11c^+^ dendritic cells and CD45^int^CD11b^+^F4/80^+^ macrophages which did, however, not significantly change over the disease course of EAE. At the same time, we observed mild accumulation of CD45^hi^ myeloid subsets in the ChP with similar kinetics as observed in SC and brain, suggesting that also inflammatory myeloid cells might use the ChP as CNS entry site.

### Numbers of CD45^+^ immune cells increase in CSF during EAE

**Fig. 4:**
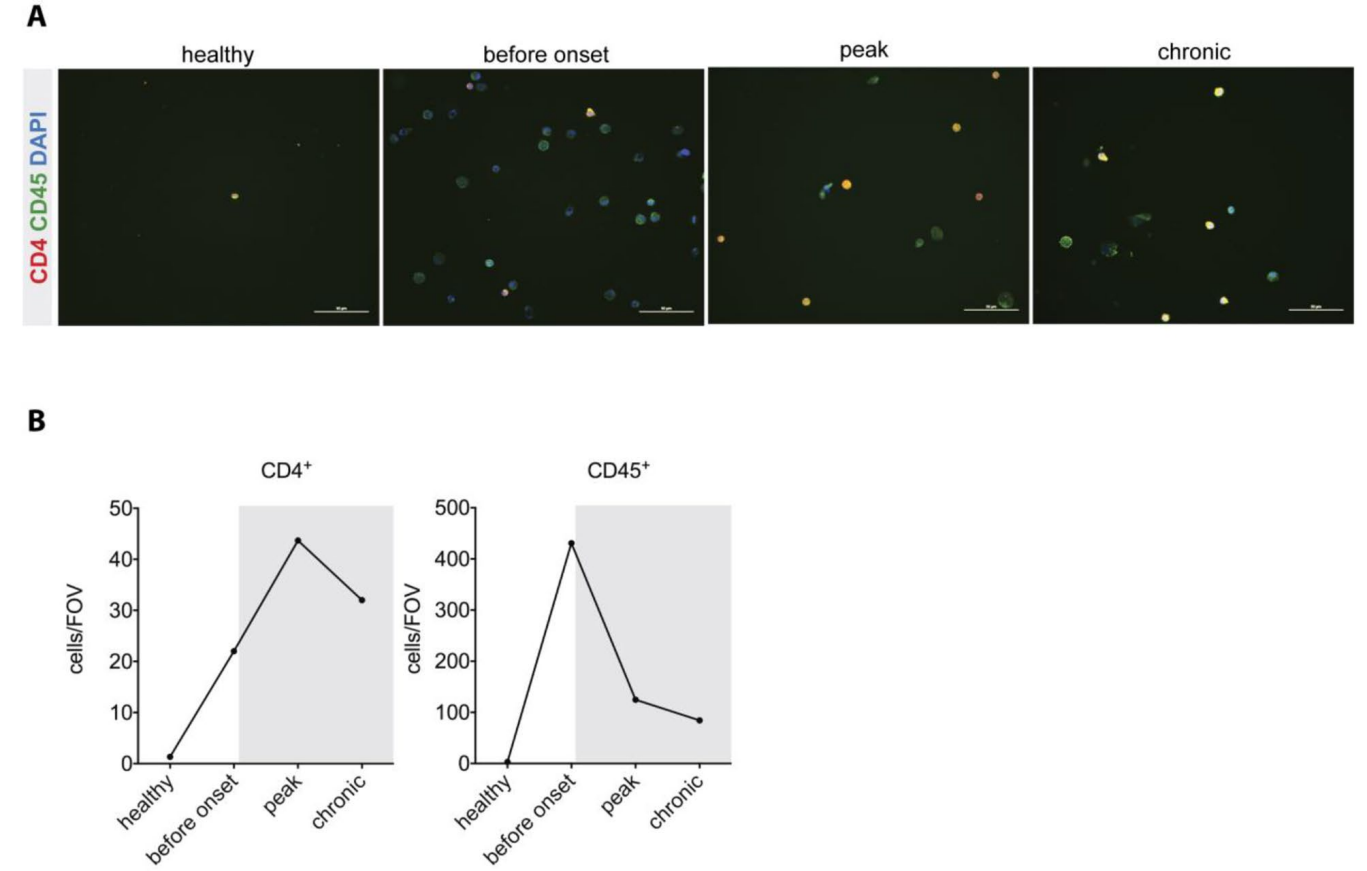
The numbers of CD45^+^ cells in the CSF increase after aEAE induction. **(A)** CSF tapping was performed by puncture of the cisterna magna of PBS perfused mice. On average a total of 15 µl of CSF was harvested and pooled from three mice, sedimented via cytospin and double-stained for CD45 (green) and CD4 (red). The CSF was collected from healthy mice and mice suffering from aEAE before onset of the disease (d 13 p.i.), at peak (d 18 p.i.) and during the chronic phase (d 30 p.i.). Scale bars = 50 μm. **(B)** Quantification of the absolute numbers of CSF derived CD45^+^CD4^+^ (yellow) and CD45^+^ (green) immune cells on the slides from healthy mice and mice suffering from aEAE.

If immune cells accumulating in the ChP during EAE crossed the ChP BCSFB and directly entered the ventricular space one would expect that increased numbers of immune cells detected in the ChP directly correlate with increased numbers of immune cells accumulating in the ventricular cerebrospinal fluid (CSF). To answer this question we collected CSF via the cisterna magna from healthy and diseased mice at different stages of EAE. Cytospin analysis detected sporadic CD45^+^ immune cells in the CSF of healthy mice (Fig. 4A, B). Before onset of aEAE, we observed a massive increase in CD45^+^ cells in the CSF, most having (multi-)lobular nuclei characteristic of granulocytes, but also increased numbers of CD45^+^CD4^+^ T cells. While at the peak of EAE, the numbers of CD45^+^ immune cells in the CSF already declined, the numbers of CD4^+^ T cells still increased starting to decline only during the chronic phase of EAE (Fig. 4A, B). The observed kinetics of the accumulation of CD45^+^ immune cells and CD45^+^CD4^+^ T cells in the CSF of mice during EAE is thus consistent with the concept that immune cells may breach the BCSFB of the ChP to enter the ventricular CSF space.

### Mouse CD4^+^ effector but not naïve T cells migrate across the pmCPECs in vitro

**Fig. 5:**
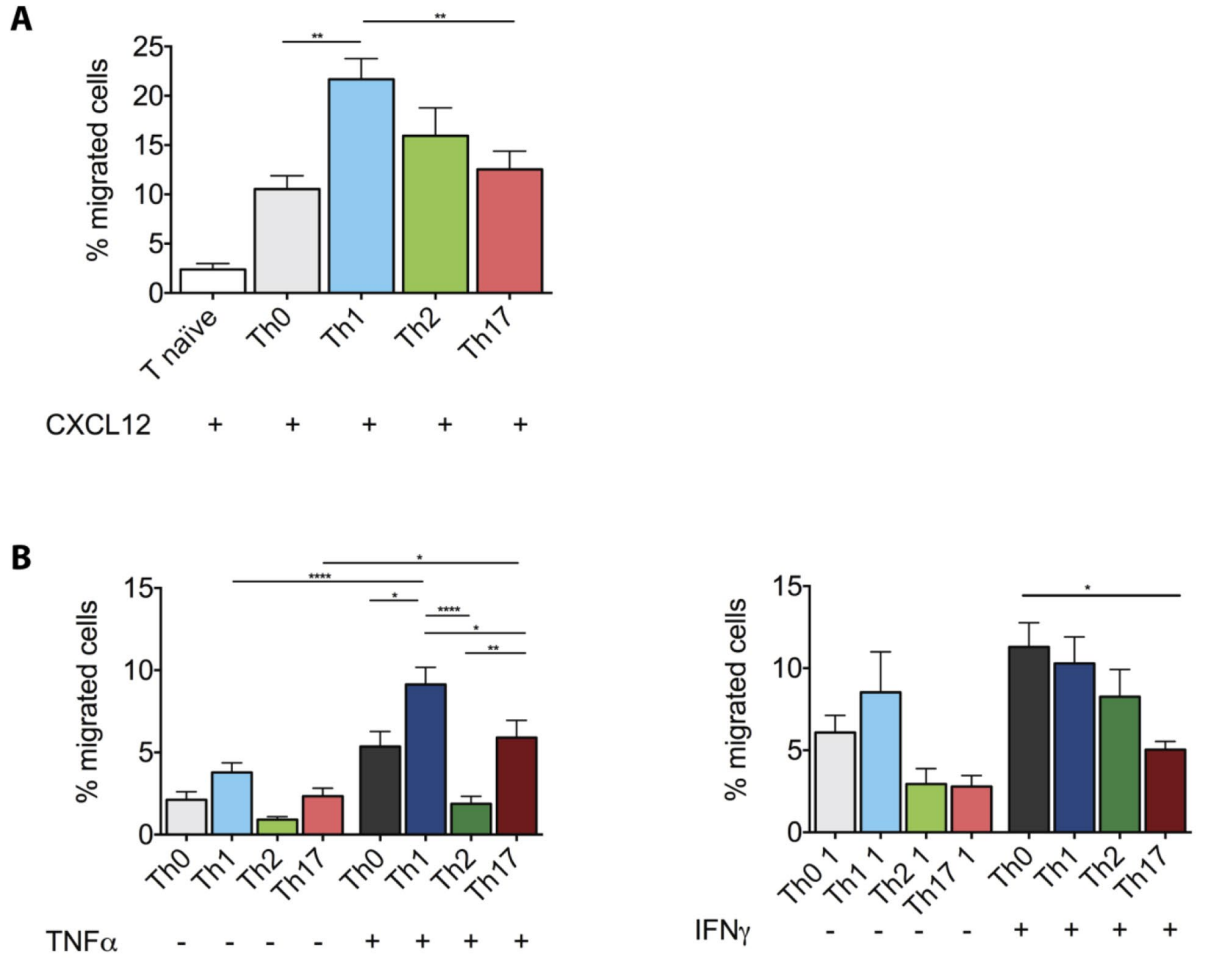
Mouse effector/memory CD4^+^ T cells can migrate across the non-stimulated and cytokine stimulated BCSFB in vitro. **(A)** The migration of MOG_35 − 55_-specific T helper cell subsets (Th0, Th1, Th2, Th17) across unstimulated pmCPEC from the basolateral to apical side towards 100 ng/mL CXCL12 after 8 h is shown. The number of T cells added to the assay (imput = 4 × 10^5^ cells) was set as 100% and the numbers of migrated T cells was assessed by flow cytometry. Bars show the mean % ± SD of two experiments with three filters per condition. **(B)** The migration of MOG_35 − 55_-specific T helper cell subsets (Th0, Th1, Th2, Th17) across unstimulated or either TNFα or IFNγ (10 ng/mL, 24 h) stimulated pmCPEC from the basolateral to apical side after 8 h is shown. The number of T cells added to the assay (imput = 4 × 10^5^ cells) was set as 100% and the numbers of migrated T cells was assessed by flow cytometry. Bars show the mean % ± SD of two experiments with three filters per condition. Statistical analysis: **(A)** one-way ANOVA and **(B)** two-way ANOVA (p < 0.05 = *, p < 0.01 = **, p < 0.001 = ***, p < 0.0001 = ****)

Although our observations that immune cells accumulate in the ChP stroma and in the ventricular CSF during progression of aEAE suggest that the ChP serves as a CNS entry site for immune cells, they do not show if the immune cells, most notably CD4^+^ T cells cross the epithelial BCSFB to reach the ventricular spaces. We have previously shown that human Th17 cells preferentially migrate across a human in vitro model of the ChP BCSFB when compared to Th1, Th2 and Th1* cells [20]. To explore if in vitro polarized mouse Th17 cells preferentially cross the BCSFB, when compared to in vitro polarized Th0, Th1 and Th2 cells, we made use of our previously established in vitro model of the mouse ChP BCSFB model consisting of primary mouse choroid plexus epithelial cells (pmCPECs) [28]. Characteristic cytokine expression profiles of the different Th cell subsets and selective expression of CCR6 on Th17 cells but no other Th cell subsets was confirmed by flow cytometry analysis as described [20], (data not shown). Mimicking CD4^+^ T-cell migration across the BCSFB in the context of CNS immune surveillance, we first compared the migration of the different CD4^+^ effector T cell subsets across non-stimulated pmCPECs from the basolateral to the apical side towards the chemokine CXCL12, which is present in the CSF under homeostatic conditions [39, 40]. Th1 cells rather than Th17 cells were most efficient in crossing pmCPEC monolayers when compared to the other CD4^+^ Th cell subsets (Fig. 5A). At the same time naïve T cells poorly crossed the pmCPEC monolayers (Fig. 5A). Under inflammatory conditions, when pmCPECs were stimulated with pro-inflammatory cytokines, e.g. TNFα or IFNγ prior to T-cell diapedesis, we observed a significant increase in the percentage of transmigrated cells in all Th cell subsets. Interestingly, even under inflammatory conditions Th1 cells rather than Th17 cells were most efficient in crossing pmCPEC monolayers when compared to the other CD4^+^ Th cell subsets (Fig. 5B).

Taken together, mouse effector CD4^+^ T cells but not naïve CD4 T cells can cross the BCSFB in vitro with Th1 cells being the most efficient T-cell subset in crossing the BCSFB under inflammatory and non-inflammatory conditions.

### Primary mouse choroid plexus epithelial cells retain in vivo characteristics of ChP epithelial cells

**Fig. 6:**
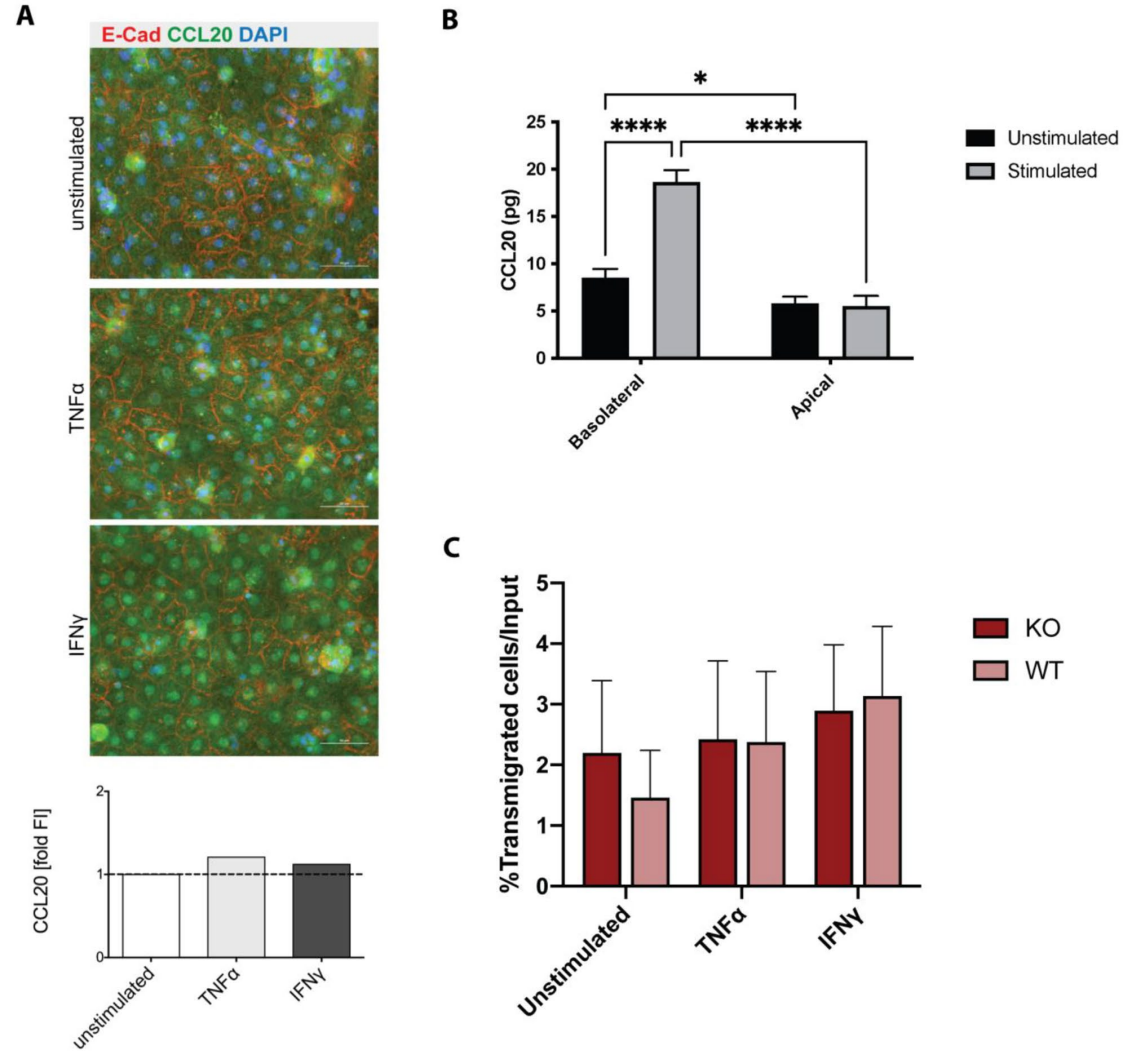
The migration of mouse Th17 cells across the BCSFB in vitro does not require CCR6. Primary mouse choroid plexus epithelial cells (pmCPECs) were used as in vitro model for the BCSFB. **(A)** Confluent monolayers of non-stimulated or TNFα or IFNγ (10 ng/mL, 16 h) stimulated pmCPECs were double-stained for CCL20 (green) and for E-Cadherin (red) and for nuclei (DAPI, blue). The bar graph shows the quantification of the fluorescence intensity for CCL20 (green) relative to unstimulated pmCPECs. Scale bars = 50 μm. **(B)** Total amount (pg) of CCL20 secretion of unstimulated and 16 h pro-inflammatory cytokine (10 ng/mL of TNFα) stimulated pmCPECs monolayers towards the basolateral (top compartment) and apical (bottom compartment) side as determined by ELISA. Bar graphs show the mean ± SD of two independent experiments measured in triplicates. **(C)** The migration of MOG_35 − 55_-specific WT or CCR6 KO Th17 cells across unstimulated or cytokine stimulated (10 ng/mL of TNFα or INFγ for 24 h prior to assay) pmCPECs from the basolateral to the apical side after 8 h is shown. The number of T cells added to the assay (imput = 4 × 10^5^ T cells) was set as 100% and the numbers of migrated T cells was assessed by flow cytometry. Bars show the mean % ± SD of 4 experiments with three filters per condition. Statistical analysis: **(B, C)** two-way ANOVA (p < 0.05 = *, p < 0.01 = **, p < 0.001 = ***, p < 0.0001 = ****). In **B** the statistical analysis is based on the combined values of the mean and the upper and lower SD.

Considering the surprising finding of Th1 rather than Th17 cells preferentially migrating across pmCPECs we next investigated the ChP epithelial cell monolayer with a specific regard to its preservation of in vivo characteristics in steady state and after stimulation with inflammatory cytokines. We confirmed constitutive apical expression of the adhesion molecules ICAM-1 and VCAM-1 on pmCPEC and their upregulated expression after pro-inflammatory cytokine stimulation (Supplementary Fig. 6A, B) as observed in vivo [18, 41]. We also confirmed constitutive expression of CCL20 by pmCPECs as observed for ChP epithelial cells in vivo [21]. Specifically, we observed strong cytoplasmic staining for CCL20 in pmCPECs, which was only slightly upregulated upon pro-inflammatory cytokine stimulation (Fig. 6A). The observed staining pattern suggested granular storage of CCL20 in pmCPECs.

We therefore next asked if CCL20 is released by pmCPECs at the apical (CSF) and/or basolateral (ChP stroma) side. To this end pmCPECs were grown on Millicell® filter inserts and stimulated or not with pro-inflammatory cytokines (TNFα). CCL20 secretion in the culture medium on both apical and basolateral sides of pmCPECs grown on Millicell® filters was determined by ELISA. Importantly, cytokine stimulation of pmCPECs did not affect their barrier properties (Supplementary Fig. 5A). Interestingly, secretion of CCL20 was polarized with significantly higher amounts of CCL20 secreted towards the basolateral side of both unstimulated and 16 h pro-inflammatory cytokine stimulated pmCPECs (8.5±0.95 pg and 18.65±1.25 pg, respectively) compared to the apical side (bottom compartment) (5.8±0.7 pg and 5.5±1.1 pg, respectively) (Fig. 6B). In addition, CCL20 levels at the basolateral side of pmCPECs were significantly higher under stimulated conditions compared to unstimulated conditions. Thus, pmCPECs retain the expression profile of trafficking molecules as observed in vivo with a polarized secretion of CCL20 especially under inflammatory conditions towards the basolateral side facing in vivo the ChP stroma.

### Migration of Th17 cells across the BCSFB in vitro does not require CCR6

As CD4^+^ Th1 cells do not express CCR6, their migration across pmCPEC monolayers must be independent of this receptor. We therefore next asked if diapedesis of Th17 cells across the BCSFB involves interaction of CCR6 with CCL20. First, we directly compared the migration of in vitro polarized 2D2 wild type (WT) or 2D2 CCR6 knockout (KO) Th17 cells across unstimulated or stimulated pmCPEC monolayers. We did not observe any significant reduction in transmigration of 2D2 CCR6 KO Th17 across pmCPECs when compared to WT 2D2 TCR Th17 cells (Fig. 6C). Thus, CCR6 is not required for the migration of mouse Th17 cells across the BCSFB.

**Fig. 7:**
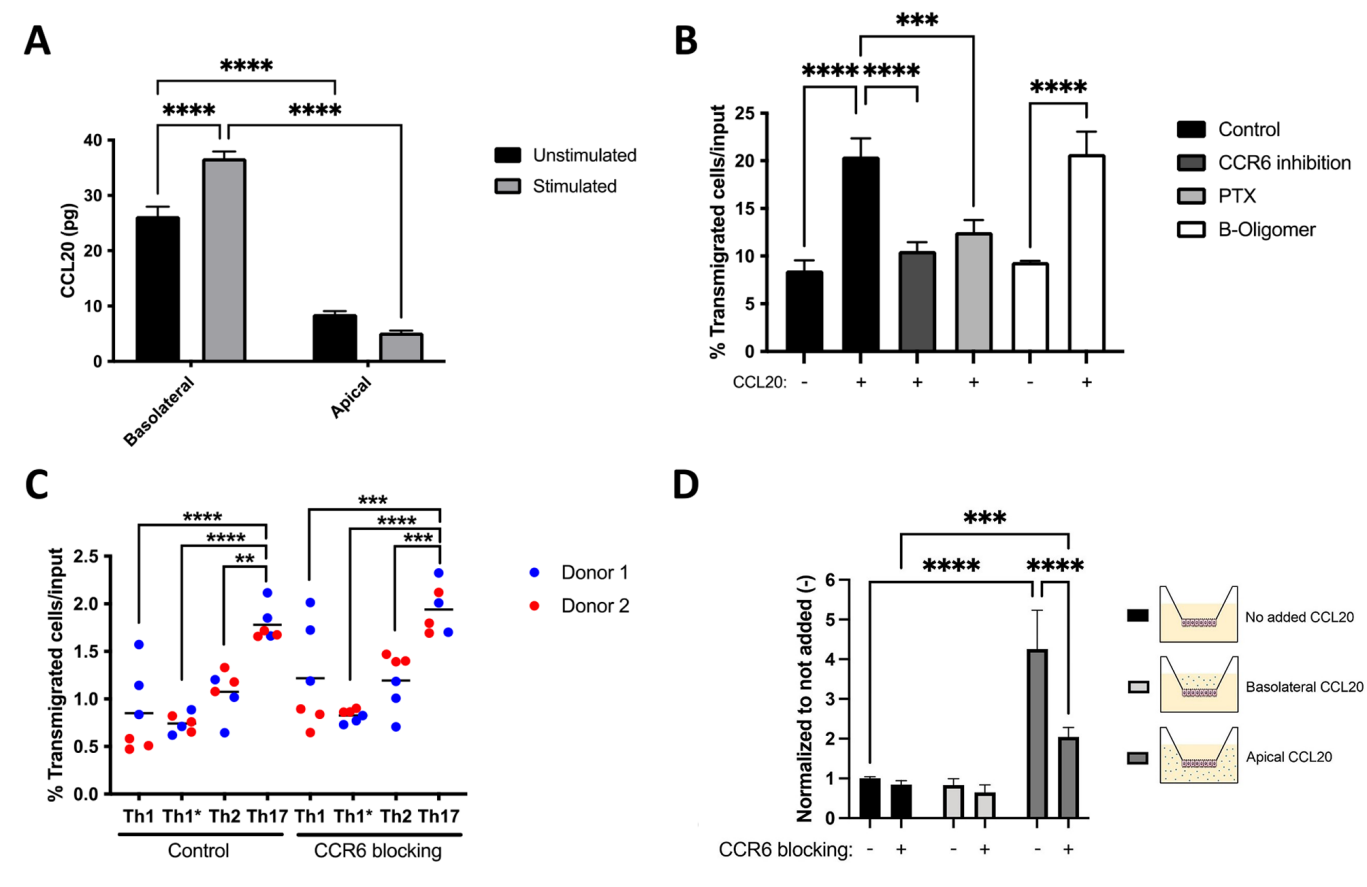
Human effector/memory Th17 cells preferentially migrate across the BSCFB but in a CCR6/CCL20 independent manner. **(A)** Total amount (pg) of CCL20 secretion towards the basolateral (top compartment) and apical (bottom compartment) side of unstimulated and 16 h pro-inflammatory cytokine (76IU/mL TNFα + 20IU/mL IFNγ) stimulated HIBCPP monolayers as determined by ELISA. Bar graph shows the mean ± SD of three independent experiments measured in triplicates. **(B)** Percentage of human Th17 cells migrated across laminin coated Millicell® filters towards the lower compartment in the presence or absence of 500 ng/mL human recombinant CCL20. Bar graph shows the mean ± SD of three independent experiments measured in triplicates. **(C)** Percentage of human Th1, Th1*, Th2 and Th17 cells that migrated from the basolateral to the apical side across 16 h pro-inflammatory cytokine (76IU/mL TNFα + 20IU/mL IFNγ)-stimulated HIBCPP monolayers after CCR6 inhibition or vehicle control treatment. Dot plot shows the mean ± SD of 2 independent experiments done in triplicates with two different human Th17-cell donors. **(D)** Percentage of human Th17 cells that migrated from the basolateral to the apical side across 16 h pro-inflammatory cytokine (76IU/mL TNFα + 20IU/mL IFNγ)-stimulated HIBCPP monolayers in the presence or absence of additional 500 ng/mL human recombinant CCL20 in the basolateral (top compartment) or apical (bottom compartment) compartment, and in the presence or absence of CCR6 inhibition and vehicle control treatment. Bar graph shows the mean ± SD of three independent experiments. Statistical analysis: **(B)** one-way ANOVA and **(A, C and D)** two-way ANOVA (*p < 0.05, **p < 0.01, ***p < 0.001 and ****p < 0.0001)

We have previously observed that human Th17 cells migrate preferentially across a human model of the BCSFB (HIBCPP cells) when compared to Th1, Th1* and Th2 cells [20]. Therefore, we next asked if CCR6 is involved in the migration of human Th17 cells across HIBCPP monolayers. First, we explored the apical and basolateral secretion of CCL20 by HIBCPP grown on Millicell® filters by ELISA. In accordance with our findings for pmCPECs we observed that HIBCPP cells secreted CCL20 in a highly polarized fashion mainly towards the basolateral side of both unstimulated and 16 h pro-inflammatory cytokine stimulated HIBCPP (26.2±1.7 pg and 36.7±1.2 pg, respectively) compared to the apical side (8.5±0.5 pg and 5.2±0.4 pg, respectively) (Fig. 7A). In addition, CCL20 levels on the basolateral side of HIBCPP were significantly higher under stimulated conditions compared to unstimulated conditions.

To next explore the role of CCR6 interaction with CCL20 in the migration of human Th17 cells across HIBCPP monolayers we made use of a CCR6 inhibitor. To this end we investigated the chemotactic migration of Th17 cells towards CCL20 across laminin coated Millicell® filters. As expected, addition of CCL20 to the lower compartment significantly increased the migration of human Th17 cells (33.2 ± 4.4%) when compared to control conditions (8.5 ± 1.0%) in the absence of CCL20 (Fig. 7B). Function blocking of CCR6 reduced the migration of Th17 cells towards CCL20 in a comparable manner as inhibiting Gαi-protein coupled receptor (GPCR)-signaling by pertussis toxin (PTX) to 10.6 ± 0.9% and 12.5 ± 1.2%, respectively, which was comparable to baseline migration (Fig. 7B). Furthermore, no impairment of Th17-cell migration was observed under B-oligomer control conditions in the presence (20.7 ± 2.3%) or absence (9.4 ± 0.1%) of CCL20 in the bottom compartment.

After confirming that human Th17 cells migrate towards CCL20 in a CCR6 dependent manner and that HIBCPP cells secrete CCL20 towards their basolateral side, we next asked if CCR6 interaction with CCL20 mediates the migration of Th17 cells across 16 h pro-inflammatory cytokine stimulated HIBCPP. Direct comparison of the migration of different effector/memory T helper subsets across HIBCPP monolayers showed in accordance with our previous observations [20] that Th17 cells had a significantly higher transmigration rate across HIBCPP monolayers when compared to Th1, Th1* and Th2 cells (Fig. 7C). However, inhibition of CCR6 did not reduce the migration of any Th cell subset across HIBCPP cells underscoring that CCR6 is not required for T-cell migration across the BCSFB. To exclude that CCL20 levels produced by HIBCPP cells in vitro are too low to mediate CCR6-dependent Th17 cell migration across this barrier we investigated Th17 migration across HIBCPP cells by adding 500 ng/mL human recombinant CCL20 to either the apical or basolateral side of the HIBCPP monolayers. Addition of CCL20 to the apical (CSF facing) side of the HIBCPP monolayer increased Th17-cell migration across HIBCPP cells in a CCR6-dependent manner while adding CCL20 to the basolateral (stroma facing) side had no effect on Th17 migration across HIBCPP cells (Fig. 7D).

Given the polarized secretion of CCL20 towards the ChP stroma facing side by our mouse and human BCSFB models these data suggest that CCL20 may rather mediate accumulation of CCR6^+^ Th17 cells into the ChP stroma than their migration across the BCSFB.

## Discussion

Our present study aimed to advance our understanding of the role of the ChP in orchestrating CNS immune cell entry in the context of neuroinflammation. Making use of EAE, an animal model of MS, we directly compared the accumulation of CD45^+^ immune cell subsets in the ChP, the brain and SC in healthy mice and from pre-clinical to chronic stages of the disease by flow cytometry. In accordance with previous observations that identified ChP resident immune cells as dendritic cells (DCs) and tissue resident myeloid cells [8, 42–44], our present study shows that compared to brain and SC, the ChP harbors higher numbers of CD45^int^ resident myeloid cells under steady state conditions. The ChP thus harbors great numbers of antigen-presenting cells (APCs) that are localized in a strategic position at the interface between the CSF and the systemic circulation and therefore provide a rampart of immune cells enforcing the ChP epithelial BCSFB function. APCs localized in the ChP stroma, between epithelial cells [42], or in case of Kolmer epiplexus cells on the apical CSF facing side of the ChP epithelial cells are positioned to efficiently sample for blood derived and CSF derived antigens [8] and may thus play a central role in shaping adaptive CNS immunity by interacting with T cells patrolling the ChP stroma.

By investigating the presence of blood derived CD45^hi^ immune cells, we here show that the ChP harbors 3 times higher fractions of CD45^hi^ immune cells when compared to brain and SC confirming that already under steady state many CD45^+^ circulating cells patrol the ChP stroma [45]. Importantly, the absolute numbers of CD45^hi^ immune cells present in the healthy ChP were comparable to the total number of CD45^hi^ cells detected in the entire healthy brain and twice as high as those detected in the SC. Amongst the CD45^hi^ immune cells we found CD4^+^ and CD8^+^ T cells in similar numbers as previously reported [46, 47] with CD4^+^ T cells outnumbering CD8^+^ T cells in addition to B220^+^ B cells. Considering the small size of the ChP, which weighs less than 5% of the brain [48], these data show that the numbers of innate and adaptive immune cells residing in the ChP have been underestimated and highlight a role of the ChP as an immunological hub within the ventricles.

It is thus plausible that the ChP also plays a major role in neuroinflammatory diseases, which is supported by several studies reporting increased numbers of immune cells in the ChP and in the CSF in a broad context of neurological disorders ranging from stroke [49, 50], SC and traumatic brain injury [51, 52], Alzheimer’s disease [53], neuropsychiatric lupus [54] to MS [55, 56] and its animal model EAE [21, 41]. While most studies were performed in animal models of neurological disorders, recent magnetic resonance imaging studies have provided direct evidence that the ChP is enlarged and inflamed in MS patients [55, 57], underscoring accumulation of immune cells in the ChP during neuroinflammation.

Our present study shows that the numbers of CD45^hi^ infiltrating immune cells and especially CD4^+^ and CD8^+^ T cells but not CD45^int^ resident immune cells in the ChP strongly increased during the development of aEAE and remained high during the chronic stages of the disease. In contrast, the numbers of CD45^hi^ infiltrating immune cells dropped in the brain and SC after the peak of EAE. The ultimate role and fate of the immune cells accumulating in the ChP during neuroinflammation still remain to be investigated. We and others have suggested that the ChP may serve as a CNS entry site for immune cells [21, 41, 49, 51, 52, 54, 58–60]. Peripherally activated T cells may in fact patrol the ChP stroma in the context of CNS immune surveillance. This is suggested by a previous study that showed that inducing peripheral inflammation by immunization of rodents with CFA omitting any CNS antigens suffices to increase the numbers of immune cells accumulating in CNS border compartments including the ChP [58]. Here we did not compare immune cell recruitment into the ChP in the absence and presence of peripheral activation of CNS-antigen specific T cells and thus our study does not allow to specify the accumulation of MOG-specific CD4^+^ T cells in the ChP after initiation of EAE. Overall our study complements, however, this previous spatiotemporal analysis of CNS immune cell infiltration comparing peripheral inflammation modeled by immunization with CFA with EAE induction induced by CNS antigens in CFA [58]. This previous study also highlighted that brain immune cell infiltration occurs via CSF-filled CNS border compartments suggesting that CSF flow pathways and thus the ChP contribute to directing immune cell infiltation into the brain during EAE.

Molecular mechanisms proposed to mediate immune cell entry into the CNS via the ChP include recruitment of P-selectin glycoprotein ligand 1 (PSGL-1) expressing CD4^+^ T cells as detected in the CSF of healthy individuals via interaction with P-selectin expressed on ChP but not CNS parenchymal vessels [10]. In addition, encephalitogenic CCR6^+^ Th17 cells were found to initiate EAE by travelling via the ChP where the epithelial cells of the BCSFB constitutively express the CCR6 ligand CCL20 [21]. Here we show that the ChP but not the brain and SC of healthy mice already harbor a high fraction of CCR6^+^CD4^+^ T cells supporting the notion of CCR6^+^CD4^+^ T cells preferentially homing to the ChP to a CCL20 rich environment. The potential role of the ChP as CNS entry site for CCR6^+^CD4^+^ T cells was underscored by the kinetics of CCR6^+^CD4^+^ T-cell accumulation observed in the brain and SC, which followed that of the ChP. Indeed, while the fraction of ChP CCR6^+^CD4^+^ T cells transiently increased to nearly 60% in the preclinical stages of EAE and subsequently decreased again prior to onset of EAE, accumulation of CCR6^+^CD4^+^ T cells in the brain and SC was only observed at later stages when the clinical disease had already started. Our study did not address the antigen-specificity of the ChP-, SC-, and brain-infiltrating CD4^+^ T cells and thus we cannot specify the precise spatiotemporal accumulation of CCR6^+^CD4^+^ MOG_35 − 55_-specific Th17 cells in the ChP, brain and SC.

Although we also detected B220^+^ B cells in the ChP of healthy mice, their numbers did not significantly increase over the disease course of EAE suggesting that ChP B cells do not play a major role in EAE pathogenesis. This is in line with recent observations identifying a large population of B cells including B lineage progenitors in the dura mater, i.e., the outer meningeal CNS border, of healthy mice and rats [61, 62]. These dura mater B cells were observed to mature upon induction of experimental neuroinflammation suggesting that the dura mater rather than the ChP may be the relevant site of B cells regulating CNS immunity in homeostasis and neuroinflammation.

The dura mater may also be a source of CNS-infiltrating myeloid cells. A recent study identified myeloid cells in the dura mater of mice that originated from the adjacent skull and vertebral bone marrow rather than the circulation [63]. Gene expression and phenotype of these dura mater myeloid cells supports their role in ensuring CNS health rather than to promote neuroinflammation. The role of the dura mater myeloid cells in providing a rampart of innate immunity at the outer BCSFB formed by the arachnoid mater versus a supply of CNS infiltrating myeloid cells will thus remain to be shown. Accordingly, the role of ChP myeloid cells in CNS immunity will need further investigations. Our present study observed a mild accumulation of CD45^hi^ myeloid subsets in the ChP with similar kinetics as observed in the brain and SC, suggesting that inflammatory myeloid cells may reach the CNS also via the ChP. At the same time, we found no change in the number of resident CD45^int^CD11b^+^CD11c^+^ dendritic cells and CD45^int^CD11b^+^F4/80^+^ macrophages. It is tempting to speculate that dura mater and ChP myeloid cells may play distinct roles in shaping adaptive immune responses that may differentially affect T-cell activation and differentiation including their trafficking properties.

Increased numbers of ChP CD45^+^ immune cells and CD45^+^CD4^+^ T cells observed in the present study preceded their increase in the CSF, which is consistent with the concept of T-cell entry into the CNS via the ChP. However, direct evidence for immune cells, specifically encephalitogenic T cells, to reach the ventricular space from the ChP stroma by crossing the epithelial BCSFB in vivo during EAE is still lacking. We have previously shown that human Th17 cells preferentially migrate from the basolateral to the apical side across a human in vitro model of the ChP BCSFB when compared to Th1, Th2 and Th1* cells [20]. In apparent contrast to these observations, we here observed that higher numbers of mouse Th1 cells when compared to Th0, Th2, Th17 and not naïve CD4^+^ T cells crossed a monolayer of pmCPECs, an in vitro model of the mouse BCSFB, from the basolateral to apical side with inflammatory conditions increasing their diapedesis rate. Although we did not test the migration of CD8^+^ T cells across the BCSFB in vitro, a previous study showed that in addition to Th1 effector cells also CD8^+^ T cells can cross the BCSFB in an inflammatory environment in vitro [64] suggesting the ChP as an entry site for cytotoxic T cells during neuroinflammation. This is in line with the our observation of an increased accumulation of also CD8^+^ T cells in the ChP of mice during EAE.

Barrier properties of pmCPECs and their suitability to study immune cell trafficking across the BCSFB were characterized before [28]. Here we confirmed that pmCPECs show apical expression of the adhesion molecules VCAM-1 and ICAM-1 as previously observed in vivo [18, 41]. Detecting protein expression of CCL20 in pmCPECs further underscored that they retain the expression profile of trafficking molecules as described in vivo [21]. Both, pmCPECs and HIBCPP cells, used as mouse and human in vitro model for the BCSFB showed polarized secretion of CCL20 towards the basolateral side facing the ChP stroma where it would be available to mediate CCR6-dependent migration of Th17 cells across the BCSFB. However, our present study shows that the migration of both, mouse and human Th17 cells across mouse and human in vitro models of the BCSFB does not require CCR6.

Given the polarized secretion of CCL20 towards the basolateral and thus ChP stroma facing side our data suggest that CCL20 may mediate accumulation of CCR6^+^ Th17 cells into or within the ChP stroma rather than their migration across the BCSFB. However, the precise migration pathway of T cells from the ChP stroma into the CNS and the molecular mechanisms involved remain to be investigated. In this context it is important to note that the neuroanatomical structure of the base of the ChP, where it folds out from the ventricular walls is not well defined [49]. It has previously been suggested that the basement membrane of the ChP epithelial cells forming the BCSFB is continuous to the parenchymal basement membrane of the superficial glia limitans surrounding the entire brain [65]. This implies that the ChP stroma is in close spatial relationship with the subarachnoid space which has recently been confirmed by an in-depth morphological study of the ChP in the mouse [66]. Thus, in contrast to crossing the epithelial BCSFB and reaching the ventricular CSF, which to this end has been considered as the main pathway of CNS T-cell entry via the ChP [67] [68], T cells may rather directly invade the subarachnoid space from the ChP stroma at the base of the ChP, where it folds out from the ventricular walls as previously suggested [49]. CNS entry of CCR6^+^ Th17 cell at this side may be guided by CCL20 deposited in the basement membrane of the ChP epithelium. Alternatively or in addition, the presence of Th17 cells within the ChP stroma could lead to the recruitment of additional immune cells into the ChP stroma from the blood or even from the CSF site [69] as IL-17 is well known to induce the secretion of multiple chemokines and pro-inflammatory cytokines exacerbating inflammation [70].

Taken together our study underscores the important role of the ChP as a potential CNS immune cell entry site and shows that CCR6 interaction with CCL20 is not required for Th17 migration across the BCSFB. It is therefore tempting to speculate that CCL20 may rather enhance Th17 cell accumulation or migration in the ChP stroma or alternatively may guide their CNS invasion along basement membranes at the base of the ChP where it folds out from the ventricular wall. The precise cellular and molecular mechanisms of T cell migration from the ChP stroma to the CNS border compartments or into the ventricular spaces remain thus to be explored. Recently developed advanced in vivo imaging technologies allowing for in vivo imaging of the mouse ChP [71] will be of fundamental importance to explore the anatomical routes of T-cell migration via the ChP into the CNS during health and neuroinflammation.

## Electronic supplementary material

Below is the link to the electronic supplementary material.

Supplementary Material 1

## Data Availability

Data – not applicable; materials are commercially available or can be made available upon request to the corresponding author.

## List of abbreviations

aEAE: active EAE
APCs: antigen presenting cells
BBB: blood-brain barrier
BCSFB: blood-cerebrospinal fluid barrier
ChP: choroid plexus
CNS: central nervous system
CSF: cerebrospinal fluid
DCs: dentritic cells
DPBS: Dulbecco’s phosphate-buffered saline
EAE: experimental autoimmune enchephalomyelitis
GPCR: G-protein coupled receptor
ICAM-1: intercellular adhesion molecule 1
LFA-1: lymphocyte function-associated antigen 1
MAM: migration assay medium
MOG: myelin oligodendrocyte glycoprotein
MS: multiple sclerosis
pmCPECs: primary mouse choroid plexus epithelial cells
PSGL-1: P-selectin glycoprotein ligand 1
RORγt: retinoid-acid-related orphan receptor γt
T_CM_: central memory T cell
T_EM_: effector memory T cell
VCAM-1: vascular cell adhesion molecule 1
WT: wild type
