# Correction to: The choroid plexus acts as an immune cell reservoir and brain entry site in experimental autoimmune encephalomyelitis

**DOI:** 10.1186/s12987-023-00457-w

**Published:** 2023-07-14

**Authors:** Ivana Lazarevic, Sasha Soldati, Josephine A. Mapunda, Henriette Rudolph, Maria Rosito, Alex Cardoso de Oliveira, Gaby Enzmann, Hideaki Nishihara, Hiroshi Ishikawa, Tobias Tenenbaum, Horst Schroten, Britta Engelhardt

**Affiliations:** 1 Theodor Kocher Institute, University of Bern, Freiestrasse 1, Bern, CH-3012 Switzerland; 2 Klinik für Kinder - und Jugendmedizin, Universitätsmedizin Mannheim, Theodor-Kutzer-Ufer 1-3, 68167 Mannheim, Germany; 3 Laboratory of Clinical Regenerative Medicine, Department of Neurosurgery, University of Tsukuba, Tsukuba, 305-8575 Ibaraki Japan; 4 Present Address: Clinic for Pediatrics and Adolescent Medicine, Johann Wolfgang Goethe University, Frankfurt/Main, Germany; 5 Present Address: Department of Physiology and Pharmacology, Sapienza University, Rome, 00185 Italy; 6 Present Address: Department of Neurotherapeutics, Yamaguchi University, Yamaguchi, 755-8505 Japan; 7 Present Address: Clinic for Pediatrics and Adolescent Medicine, Sana Clinic Lichtenberg, Charité, Berlin, Germany

**Correction**: ***Fluids Barriers CNS*20, 39 (2023)**

10.1186/s12987-023-00441-4

Following publication of this article [1], the author group became aware of a labelling error in the legend of Fig. 7D, where the gray scales of the conditions showing apical and basolateral application of CCL20 were reversed.

The correct figure should have appeared as shown below.

**Figure 1:**
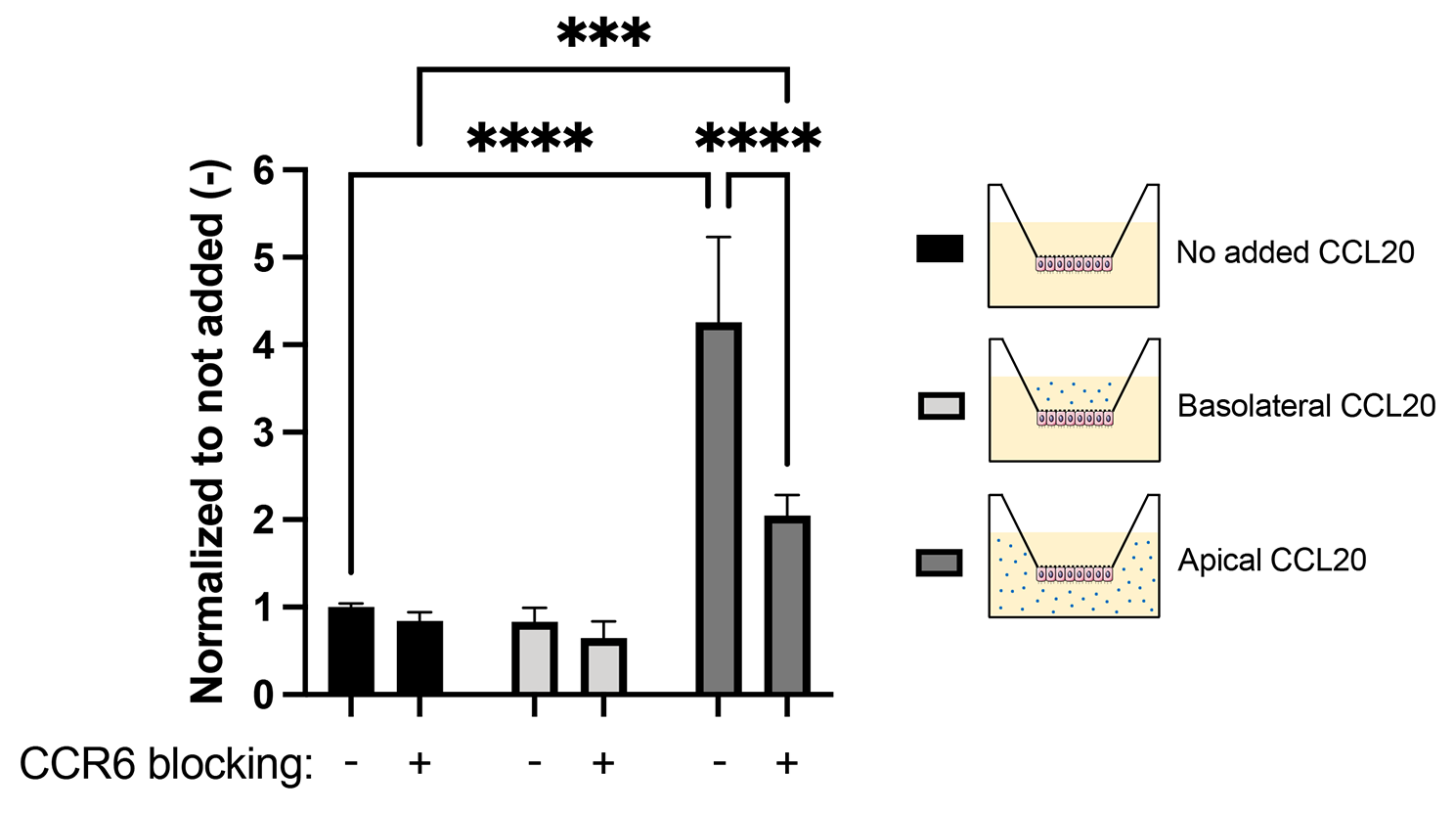
**Figure 7D **

The original article has been corrected.
