# Supplementary material for: The choroid plexus acts as an immune cell reservoir and brain entry site in experimental autoimmune encephalomyelitis
Source: Fluids Barriers CNS. 2023 Jun 1;20:39. doi: 10.1186/s12987-023-00441-4 (PMC10236715; doi:10.1186/s12987-023-00441-4)
Supplement: Supplementary file 1 — Supplementary Material 1 [file 12987_2023_441_MOESM1_ESM.pdf]

Supplementary Information – Figures and Tables

The choroid plexus acts as an immune cell reservoir and brain entry site in experimental autoimmune encephalomyelitis

Ivana Lazarevic[1†], Sasha Soldati[1†], Josephine A. Mapunda[1†], Henriette Rudolph[2,4], Maria Rosito[1,5], Alex Cardoso de Oliveira[1], Gaby Enzmann[1], Hideaki Nishihara[1,6], Hiroshi Ishikawa[3], Tobias Tenenbaum[2,7], Horst Schroten[2] and Britta Engelhardt[1]

A

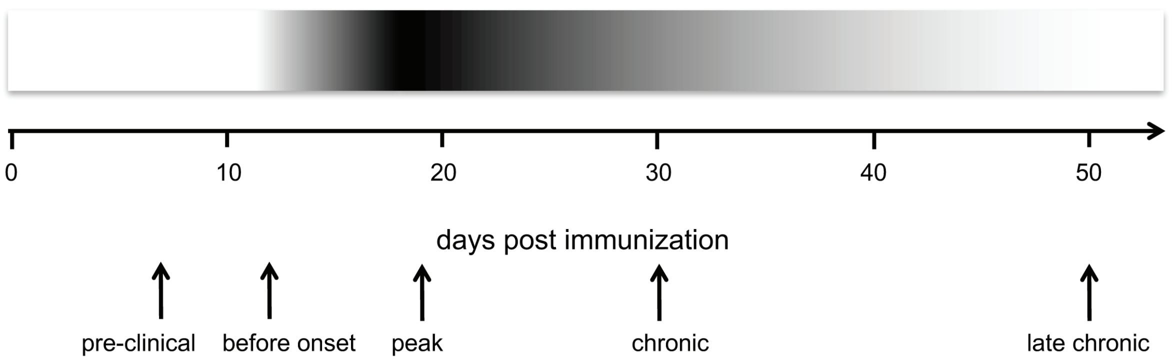

B

	healthy	pre-clinical<br>(no signs of disease)	before onset<br>(start of weight loss)	peak	chronic	late chronic
Days post immunization		8-9	11-13	16-20	30	50
N° of experiments x N° of mice	5 x 5	5 x 5	4 x 5	1 x 4<br>5 x 5	1 x 3<br>1 x 4<br>2 x 5	1 x 4<br>2 x 5
Total N° of analyzed mice	25	25	20	29	27	14
Mean ± SD of EAE severity (score)	0	0	0	1.8 ± 0.4	0.6 ± 0.6	1.2 ± 0.4

Figure S1. Experimental design of EAE experiments. (A) Timeline of the aEAE experiments with day 0 = day of EAE induction by immunization with MOG_{35-55} in CFA and spanning to day 50 post immunization. Arrows indicate the time-points and assigned names of harvesting immune cells from the brain, the spinal cord and choroid plexus. **(B)** Overview of the number of individual experiments and mice investigated. Immune cells were isolated from three-five mice per time point and analyzed by flow cytometry. A total of six independent aEAE experiments were performed. Cells were isolated at pre-defined time-points post immunization, i.e., pre-clinical (days 8-9 p.i.), before onset (days11-13 p.i.), at the peak of the disease (days 16-20 p.i.), and during the chronic (day 30 p.i.) and late chronic phase (day 50 p.i.).

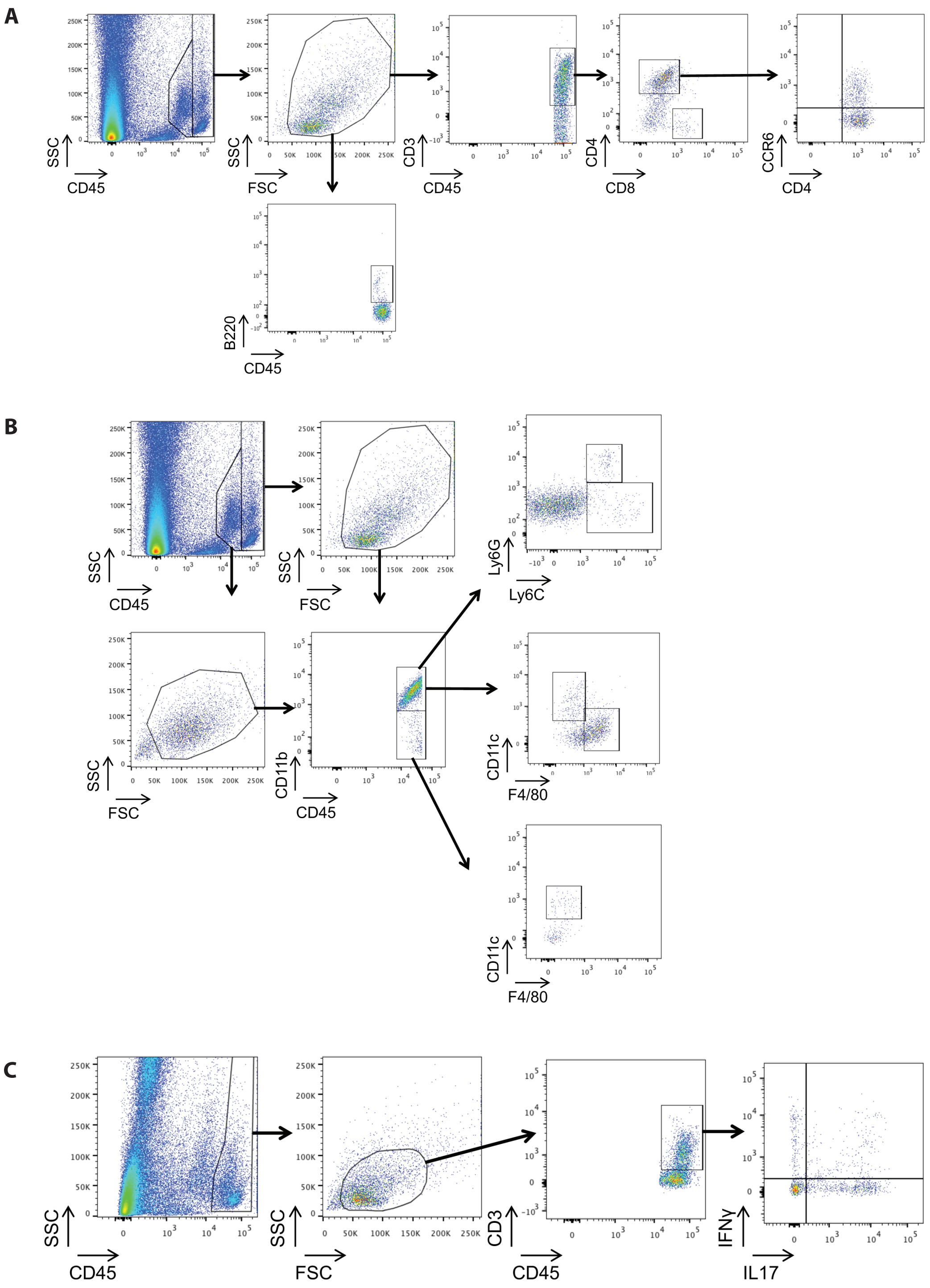

Figure S2. Gating strategy for flow cytometry analysis. Gating strategy examples (here ChP) for **(A)** $CD45^{hi}$ T and B cells and **(B)** $CD45^{int}$ resident and $CD45^{hi}$ infiltrating myeloid cells using cell surface markers as indicated. **(C)** Gating strategy (here ChP) for intracellular T cell cytokine stainings as indicated.

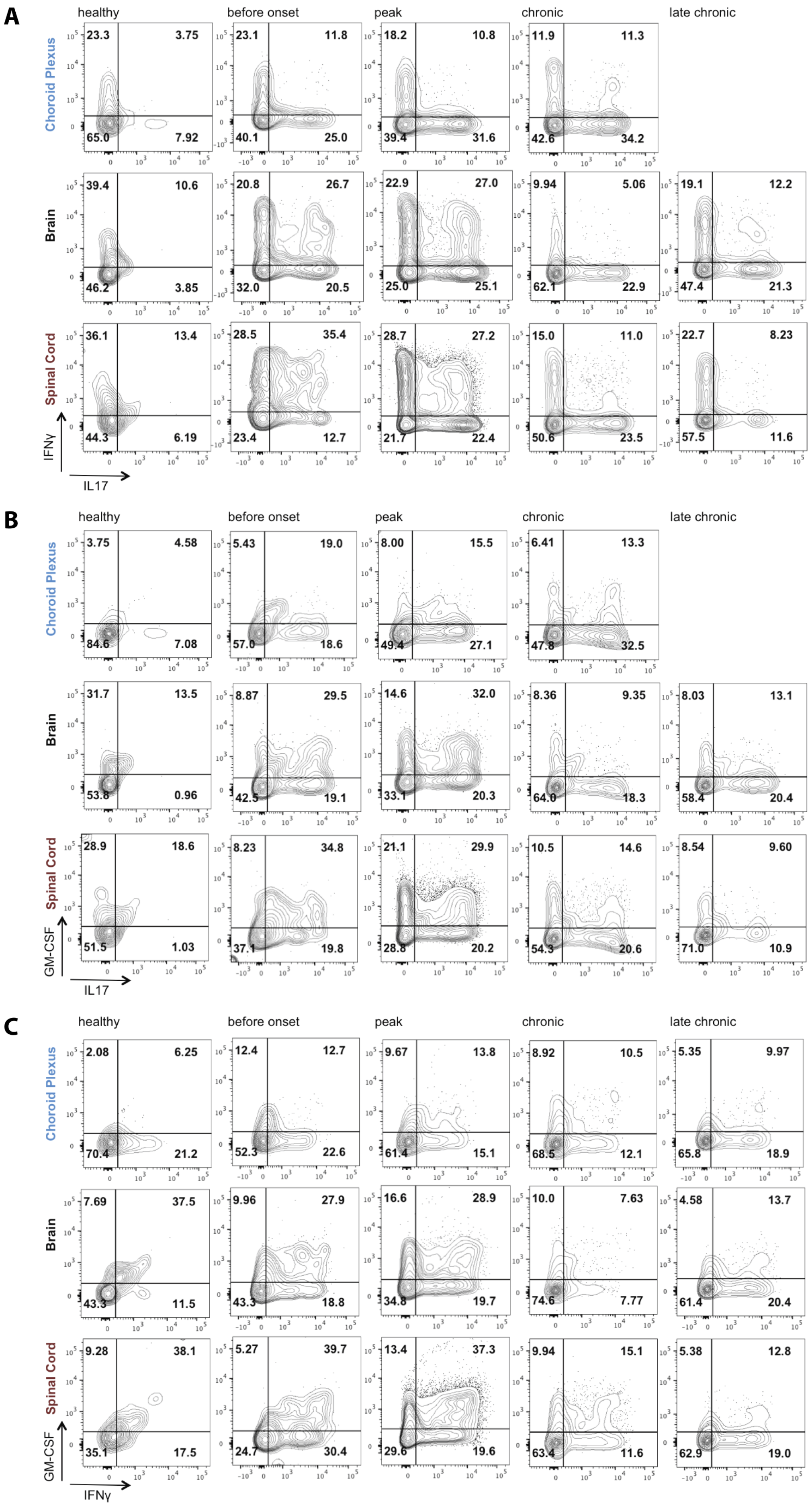
A
healthy
before onset
peak
chronic
late chronic
Choroid Plexus
23.3 3.75 65.0 7.92
23.1 11.8 40.1 25.0
18.2 10.8 39.4 31.6
11.9 11.3 42.6 34.2
Brain
39.4 10.6 46.2 3.85
20.8 26.7 32.0 20.5
22.9 27.0 25.0 25.1
9.94 5.06 62.1 22.9
19.1 12.2 47.4 21.3
Spinal Cord
36.1 13.4 44.3 6.19
28.5 35.4 23.4 12.7
28.7 27.2 21.7 22.4
15.0 11.0 50.6 23.5
22.7 8.23 57.5 11.6
IFNγ
IL17
B
healthy
before onset
peak
chronic
late chronic
Choroid Plexus
3.75 4.58 84.6 7.08
5.43 19.0 57.0 18.6
8.00 15.5 49.4 27.1
6.41 13.3 47.8 32.5
Brain
31.7 13.5 53.8 0.96
8.87 29.5 42.5 19.1
14.6 32.0 33.1 20.3
8.36 9.35 64.0 18.3
8.03 13.1 58.4 20.4
Spinal Cord
28.9 18.6 51.5 1.03
8.23 34.8 37.1 19.8
21.1 29.9 28.8 20.2
10.5 14.6 54.3 20.6
8.54 9.60 71.0 10.9
GM-CSF
IL17
C
healthy
before onset
peak
chronic
late chronic
Choroid Plexus
2.08 6.25 70.4 21.2
12.4 12.7 52.3 22.6
9.67 13.8 61.4 15.1
8.92 10.5 68.5 12.1
5.35 9.97 65.8 18.9
Brain
7.69 37.5 43.3 11.5
9.96 27.9 43.3 18.8
16.6 28.9 34.8 19.7
10.0 7.63 74.6 7.77
4.58 13.7 61.4 20.4
Spinal Cord
9.28 38.1 35.1 17.5
5.27 39.7 24.7 30.4
13.4 37.3 29.6 19.6
9.94 15.1 63.4 11.6
5.38 12.8 62.9 19.0
GM-CSF
IFNγ

Figure S3. Accumulation of CD3$^+$ CD4$^+$T cell subsets in the ChP, brain and SC during EAE. (A, B, C) Detection of signature cytokines characteristic for Th1 (IFNγ), Th17 (IL17), Th1* (IFNγ and IL17) and encephalitogenic CD4$^+$ T cells (IFNγ and GM-CSF or IL-17 and GM-CSF) was determined by flow cytometry on immune cells isolated from the ChP, brain and spinal cord of healthy mice and mice at the indicated timepoints after induction of aEAE. Data are shown as contour plots, numbers indicate the % of cells in each quadrant.

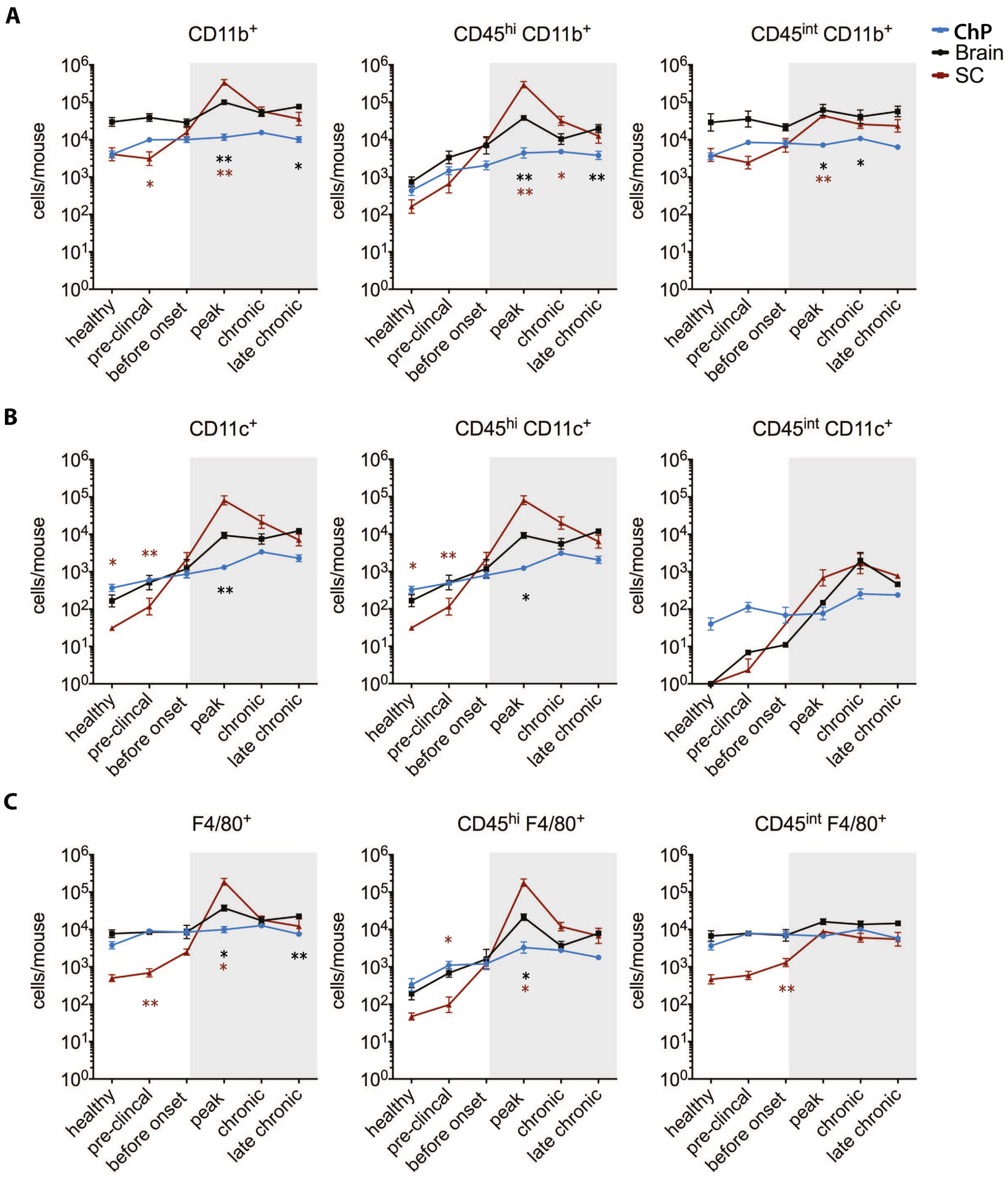

Figure S4. Numbers of myeloid cell subsets in the choroid plexus, brain, and spinal cord of healthy C57BL/6J mice and during aEAE progression. Absolute numbers of myeloid cells per mouse in the choroid plexus (ChP), brain and spinal cord (SC) of healthy mice and mice suffering from aEAE as acquired by flow cytometry are shown. The $CD11b^{+}$ **(A)**, $CD11c^{+}$ **(B)**, and the $F4/80^{+}$ cells **(C)** were further differentiated into $CD45^{hi}$ and $CD45^{int}$ subsets. The graphs show means ± SEM of six independent experiments. Significant differences between ChP and SC or ChP and brain are shown in brown and black stars, respectively. Statistical analysis: **(A-C)** one-way ANOVA ($p < 0.05$ = *, $p < 0.01$ = **, $p < 0.001$ = ***, $p < 0.0001$ = ****).

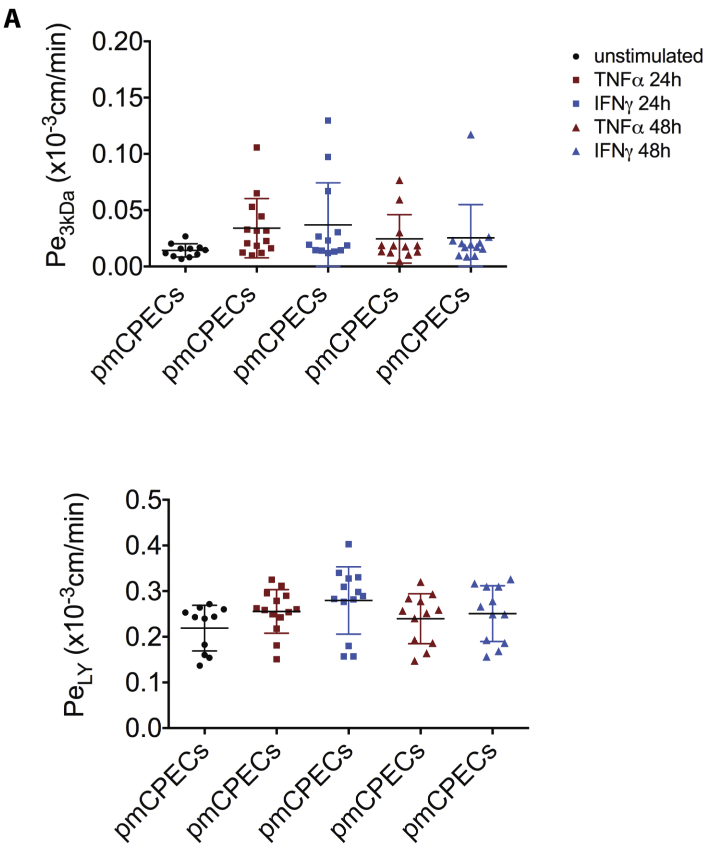

Figure S5. Primary mouse choroid plexus epithelial cells (pmCPECs) establish a tight barrier under stimulated and cytokine-stimulated conditions. The permeability coefficients (Pe) of the pmCPECs for 3 kDa dextran (Pe_{3kDa}) (top) and Lucifer Yellow (bottom) were measured across unstimulated and 24hours or 48 hours cytokine (10 ng/mL of TNFα or 100 U/mL of IFNγ) stimulated pmCPEC monolayers. Data represent mean ± SD of three independent experiments with 3 to 4 filters per condition.

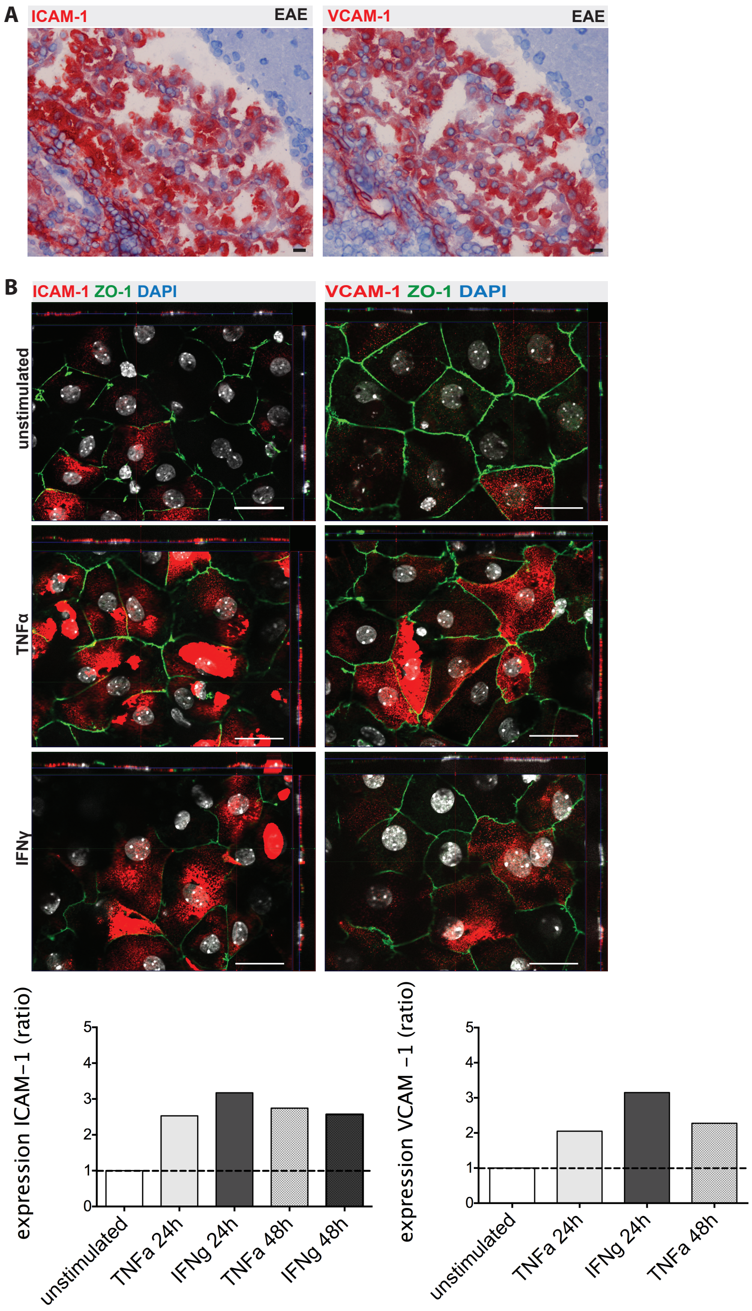

Figure S6. Primary mouse choroid plexus epithelial cells (pmCPECs) express ICAM-1, VCAM-1, and CCL20. (A) Immunostaining for ICAM-1 and VCAM-1 as detected on the ChP in the lateral ventricle in mouse brain cryosections obtained from C57BL/6J mice during EAE. Peroxidase staining, Hemalaun counterstaining. Scale bars = 20 µm. **(B)** Representative orthographic projections of pmCPECs layers immunostained for the adhesion molecules ICAM-1 (red) or VCAM-1 (red) in combination with the junctional protein ZO-1 (green) and for nuclei (DAPI, white) in the absence or after 24 hours stimulation with 10 ng/mL of TNFα and 100 U/mL of IFNγ. Immunostaining for ICAM-1 and VCAM-1 on stimulated versus non-stimulated pmCPECs was quantified by on-cell western and is presented as ratio relative to unstimulated pmCPECs. Scale bars = 50 µm.

Supplementary Table 1. List of employed antibodies for flow cytometry, immunofluorescence, immunohistochemistry, and on-cell western.

Antibodies for flow cytometry

Primary Antibodies	clone	Company	CAT Nr.	Working Conc.
rat anti- Mouse CD3 - AF700	17A2	Pharmingen BD Biosciences	561388	1 to 200
rat anti-mouse CD4 - FITC	RM4-5	BioLegend	100509	1 to 100
rat anti-mouseCD8α - PerCP	53-6.7	BioLegend	100731	1 to 100
rat anti-mouse CD19 - Pacific Blue	6D5	BioLegend	115526	1 to 100
armenian hamster anti-mouse CD11c - PE	HL3	Pharmingen BD Biosciences	557401	1 to 100
rat anti-mouse CD11b - Pacific Blue	M1/70	Biolegend	101223	1 to 100
rat anti-mouse CD45 - APC	30-F11	BioLegend	103112	1 to 1000
rat anti-mouse CD45 - PE/Cy7	30-F11	BioLegend	103114	1 to 1000
rat anti-mouse CD45R/B220 - AF488	RA3-6B2	Pharmingen BD Biosciences	557669	1 to 100
rat anti-mouse F4/80 - PerCP	BM8	BioLegend	123125	1 to 100
rat anti-mouse Ly6G - FITC	1A8	BioLegend	127605	1 to 500
rat anti- mouse Ly6C - AF700	HK1.4	BioLegend	128023	1 to 500
rat anti-mouse IFNγ - PE	XMG1.2	Pharmingen BD Biosciences	554412	1 to 40
rat anti-mouse GM-CSF - FITC	MP1-22E9	BioLegend	505404	1 to 40
rat anti-mouse IL4 - APC	11B11	BioLegend	504105	1 to 40
rat anti mouse IL17A - Pacific Blue	TC11-18H10.1	BioLegend	506918	1 to 40
armenian hamster anti CCR6 - PE	29-2L17	BioLegend	129804	1 to 100
rat IgG1 - PE	R3-34	Pharmingen BD Biosciences	559318	1 to 40
rat IgG1 - APC	R3-34	Pharmingen BD Biosciences	554686	1 to 40
rat IgG1 - Pacific Blue	RTK2071	Biolegend	400419	1 to 40
rat IgG2a -FITC	R35-95	Pharmingen BD Biosciences	555843	1 to 100
rat IgG2a - PE	RTK2758	BioLegend	400507	1 to 100
rat IgG2b - APC	RTK4530	BioLegend	400611	1 to 100
rat IgG2b - Pacific Blue	RTK4530	BioLegend	400627	1 to 100
rat IgG2b - PE/Cy7	RTK4530	BioLegend	400618	1 to 100
rat IgG2b - AF700	RTK4530	BioLegend	400628	1 to 200
rat IgG2a - PerCP	RTK2758	BioLegend	400529	1 to 100
rat IgG2b - FITC	RTK4530	BioLegend	400633	1 to 100
rat IgG2b - PerCP	A95-1	Pharmingen BD Biosciences	552991	1 to 100
Armenian hamsterIgG - PE	HTK888	BioLegend	400907	1 to 100

Antibodies for immunofluorescence, immunohistology, on-cell western

Primary Antibodies	clone	Company	CAT Nr.	Working Conc.
rat anti-mouse ICAM-1	25ZC7	hybridoma supernatant	in house	undiluted
rat anti-mouse VCAM-1	9DB3	hybridoma supernatant	in house	undiluted
rat anti-human CD44; used as isotype control	9B5	hybridoma supernatant	in house	undiluted
polyclonal rabbit anti-mouse CCL20	N.A.	ABCAM	ab9829	10 µg/mL
rat anti-mouse CD4	GK1.5	hybidoma supernatant	in house	undiluted
mouse anti-human E-Cadherin	Clone 36/E-Cadherin	BD Biosciences	610182	10 µg/mL
rat anti-mouse CD45 - Alexa 488	30-F11	Biologend	103122	10 µg/mL
polyclonal rabbit anti mouse ZO-1	N.A.	Thermo Fisher Scientific	61-7300	5 µg/mL

Secondary Antibodies	clone	Company	CAT Nr.	Working Conc.
polyclonal donkey anti-mouse IgG - Cy3	N.A.	Jackson Immuno Research	715-165-151	2.5 µg/mL
polyclonal goat anti-rabbit IgG - Alexa488	N.A.	Invitrogen	A32731	10 µg/mL
polyclonal donkey anti-rat IgG - Cy3	N.A.	Jackson Immuno Research	712-165-150	7 µg/mL
